# From the Tunnels into the Treetops: New Lineages of Black Yeasts from Biofilm in the Stockholm Metro System and Their Relatives among Ant-Associated Fungi in the Chaetothyriales

**DOI:** 10.1371/journal.pone.0163396

**Published:** 2016-10-12

**Authors:** Martina Réblová, Vit Hubka, Olle Thureborn, Johannes Lundberg, Therese Sallstedt, Mats Wedin, Magnus Ivarsson

**Affiliations:** 1 Department of Taxonomy, Institute of Botany of the Czech Academy of Sciences, 252 43, Průhonice, Czech Republic; 2 Department of Botany, Faculty of Science, Charles University in Prague, 128 01, Prague, 2, Czech Republic; 3 Laboratory of Fungal Genetics and Metabolism, Institute of Microbiology of the Czech Academy of Sciences, 142 20, Prague, 4, Czech Republic; 4 Department of Ecology, Environment and Plant Sciences, Stockholm University, 106 91, Stockholm, Sweden; 5 Department of Botany, Swedish Museum of Natural History, 104 05, Stockholm, Sweden; 6 Department of Biology, University of Southern Denmark, 5230, Odense, Denmark; 7 Department of Palaeobiology, Swedish Museum of Natural History, 104 05, Stockholm, Sweden; University of California Riverside, UNITED STATES

## Abstract

Rock-inhabiting fungi harbour species-rich, poorly differentiated, extremophilic taxa of polyphyletic origin. Their closest relatives are often well-known species from various biotopes with significant pathogenic potential. Speleothems represent a unique rock-dwelling habitat, whose mycobiota are largely unexplored. Isolation of fungi from speleothem biofilm covering bare granite walls in the Kungsträdgården metro station in Stockholm yielded axenic cultures of two distinct black yeast morphotypes. Phylogenetic analyses of DNA sequences from six nuclear loci, ITS, nuc18S and nuc28S rDNA, *rpb*1, *rpb*2 and β-tubulin, support their placement in the Chaetothyriales (Ascomycota). They are described as a new genus *Bacillicladium* with the type species *B*. *lobatum*, and a new species *Bradymyces graniticola*. *Bacillicladium* is distantly related to the known five chaetothyrialean families and is unique in the Chaetothyriales by variable morphology showing hyphal, meristematic and yeast-like growth *in vitro*. The nearest relatives of *Bacillicladium* are recruited among fungi isolated from cardboard-like construction material produced by arboricolous non-attine ants. Their sister relationship is weakly supported by the Maximum likelihood analysis, but strongly supported by Bayesian inference. The genus *Bradymyces* is placed amidst members of the Trichomeriaceae and is ecologically undefined; it includes an opportunistic animal pathogen while two other species inhabit rock surfaces. ITS rDNA sequences of three species accepted in *Bradymyces* and other undescribed species and environmental samples were subjected to phylogenetic analysis and in-depth comparative analysis of ITS1 and ITS2 secondary structures in order to study their intraspecific variability. Compensatory base change criterion in the ITS2 secondary structure supported delimitation of species in *Bradymyces*, which manifest a limited number of phenotypic features useful for species recognition. The role of fungi in the speleothem biofilm and relationships of *Bacillicladium* and *Bradymyces* with other members of the Chaetothyriales are discussed.

## Introduction

Fungi from rock-dwelling habitats can endure extreme conditions; they are of polyphyletic origin, apparently lack sexual reproductive structures, are largely undescribed and usually figure under strain or sample numbers in large-scale multilocus phylogenetic analyses. Their closest relatives are often well-described taxa from various biotopes, but also other unnamed strains or whole lineages of such organisms thriving in the most unlikely places [1–4]. During our research of the Stockholm metro system we primarily focused on fungi classified in the Chaetothyriales living in the biofilm covering bare granite walls of the artificial cave of the Kungsträdgården metro station.

The order Chaetothyriales [5] harbours non-lichenised, mostly melanised ascomycetes with immense ecological diversity and morphological variability, so far accommodated in five families, i.e., the Chaetothyriaceae [6], Cyphellophoraceae [7], Epibryaceae [8], Herpotrichiellaceae [9], Strelitzianaceae [10] and Trichomeriaceae [11]. Members of the Chaetothyriales, often referred to as black yeasts, play a diverse role in nature. They act as saprobes, biotrophs and weak plant pathogens, and also opportunistic pathogens, some of which can cause fatal infections in humans and animals. They include lichenicolous, bryophilous, epilithic and endolithic taxa. They also occur in soil, resin, and nutrient-poor substrates or humid indoor environments such as bathroom surfaces and washing machines [12, 13]. Numerous undescribed and morphologically poorly differentiated chaetothyrialean fungi isolated from nests or tunnel-shaped runway galleries of tropical ants were discovered recently and placed in the Chaetothyriales with the aid of molecular data [14–17].

The umbrella term ‘black yeasts’ was coined by Ulson for a group of yeast-like organisms unrelated to the true yeasts (Saccharomycetes), which caught the attention of medical mycologists for their pathogenic capacity to humans and animals [18]. The group comprises microscopic fungi with pigmented mycelium that can reproduce by budding cells, or more often demonstrate mycelial growth and produce conidia from phialides, annellides or undifferentiated conidiogenous cells, or exhibit meristematic growth with in situ microcolonial growth pattern [19–24]. The presence of melanin in cells improves their resistance to UV irradiation and survival in extreme, nutritionally poor or toxic environments.

Chaetothyrialean fungi, one of the black yeast orders, inhabiting plant or stone surfaces under stressful and extreme conditions are usually characterised by compact colonies, slow growth, heavily melanised mycelium and predominantly asexual reproduction [1–3, 25–28]. Apart from natural occurrence on exposed surfaces of rocks, they are responsible for the bio-pitting phenomena [29] and have a potential role in biodeterioration of stone monuments, outdoor sculptures and archaeological objects [1, 30–36]. According to the molecular data, these predominantly rock-inhabiting fungi are accommodated in several different orders of the Pezizomycotina, among which the Chaetothyriales are prominent, where they form ecologically specialized lineages. Moreover, ancestral state reconstructions in the Chaetothyriales suggest that rock-inhabiting fungi are ancestral to opportunistic pathogens based on the evidence of their early diverging lineages [3].

Speleothems, secondary mineralisations in caves, are known as vital microbial habitats many times formed by the presence of its inhabitants [37–43]. Depending on the geochemical conditions and exposure to light, speleothem and cave-wall biofilms host various microorganisms such as bacteria, cyanobacteria, algae (diatoms), fungi, testate amoebae, but also collembolans, mites and even spiders and other larger invertebrates [43–47]. Speleothem formation in magmatic rock environments is usually cyclic with repeated layers of biofilms and inorganic mineralisation of carbonates or opal-A. As the speleothems are mineralized the biofilms are preserved as organic layers, comprising encrusted fungal filaments, bacterial remnants and coccoid cell-like structures [47]. The microbial influence on speleothem genesis was investigated in the granite/dolerite hosted Tjuv-Ante’s cave in Västerbotten County, northern Sweden. Sallstedt et al. [47] confirmed a connection between speleothem biofilm of bacterial communities dominated by Actinobacteria and speleothem formation. Fungal communities that appeared to be secondary colonizers acted as both constructive and destructive agents of the primary mineral, and thus enhanced speleothem creation, contributing to the formation of coralloid morphotypes.

The Kungsträdgården metro station in Stockholm is an urban, artificial environment constructed in granite and is located underground. The platform is permanently lit by fluorescent lamps and is one of few stations with exposed walls of ‘Stockholm granite’ [48]. Due to the unusual environmental conditions such as naked rock walls, running meteoritic water and artificial light, the station is hosting a unique ecosystem on the platform walls far from being investigated and understood. The most prominent are active fungal and diatom communities, both involved in carbonate mineralisation, speleothem formation and destruction [45, 46].

During our research of fungal communities in the granitic Kungsträdgården metro station a sample of the biofilm was collected and subjected to DNA extraction and sequencing. The specimen of biofilm was then cultivated on nutrient media and several isolates of two different black yeast morphotypes were obtained in axenic culture. Both morphotypes shared some features with other rock-inhabiting fungi, i.e., slow-growing melanised colonies, vegetative mycelium consisting mostly of moniliform hyphae growing through enteroblastic proliferation and the tendency of shifting to meristematic development. In addition, the first morphotype represented by two strains showed, at first, wet colonies becoming crustose and velvety with time, the maximum growth temperature was 30°C, the micromorphology was typical by the production of budding yeast cells, torulose hyphae and pseudoparenchyma. The second phenotype involving six strains was characterised by velvety colonies, production of endoconidia and inability to grow at 28°C. Preliminary analyses of DNA sequences of the two undescribed black yeasts revealed their close relationship with members of the Chaetothyriales. The first morphotype was distantly related to the known chaetothyrialean families while the second unknown fungus showed affinity with *Bradymyces* Hubka et al., a member of the Trichomeriaceae [49]. The originally monotypic Trichomeriaceae was introduced [11] for foliar epiphytes or saprobes on honey dew insect excretions resembling sooty moulds and developing superficial ascomata with fissitunicate asci. In this family the numbers of included taxa are most rapidly growing in the Chaetothyriales and to date it comprises species classified in more than ten genera and several unnamed lineages of rock-inhabiting [3, 8, 28] and ant-associated fungi [16, 17], which could not have been differentiated before the availability of molecular tools.

The aim of this study is to investigate phylogenetic relationships of the two unidentified black yeasts with members of the Chaetothyriales utilising six nuclear loci in three phylogenetic analyses. In order to study intraspecific relationships at the RNA structure level among described species of *Bradymyces* and other related isolates, we used the CBC (compensatory base change) criterion in the ITS2 secondary structure. The CBC concept correlates with Mayr’s biological species concept and has been used to delimit species [50–52]. It is based on co-evolution of nucleotides involved in the double-sided substitution in two most conserved helices II and III of the ITS2 molecule. We performed in-depth comparative analyses of ITS1 and ITS2 secondary (2D) structures and mapped all existing substitutions onto the predicted 2D models of ITS1 and ITS2 and onto the ITS phylogram.

## Materials and Methods

### Setting and Sampling

**Fig 1:**
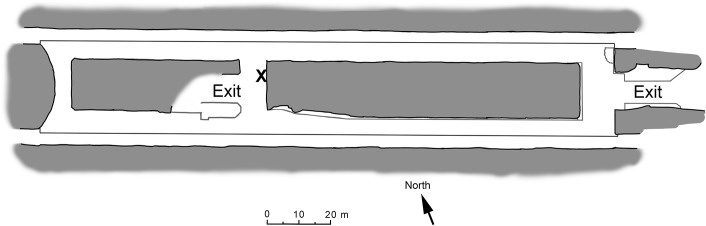
An overview map of the Kungsträdgården metro station in Stockholm. X marks the sample location.

**Fig 2:**
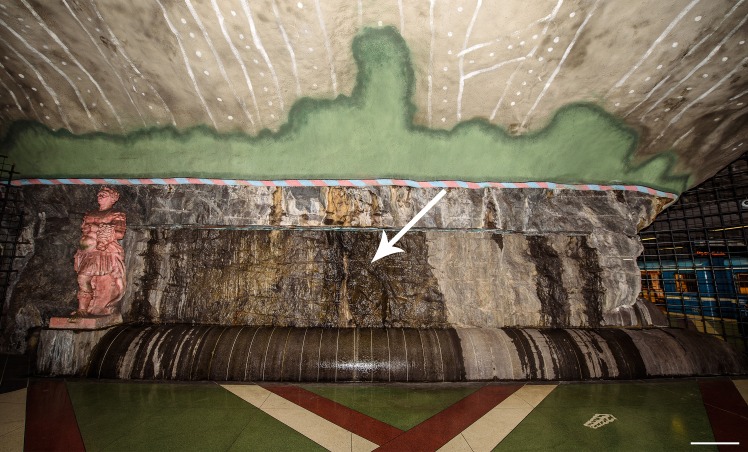
The Kungsträdgården metro station in Stockholm. Overview of the granite wall covered in fungal biofilm. Colorations represent wet and dry biofilm or mineral precipitates. Arrow indicates wall with wet biofilm and sample location. Bar = 1 m.

The Kungsträdgården metro station belongs to the line 10 and 11 of the Stockholm metro system and was opened in 1977. The station is constructed in ‘Stockholm granite’ whose approximate age is 1.8 Ga [48], and is located at a depth of 34 m below ground and 29.3 m below sea level [45]. The exits are located about 150 meters from the Baltic Sea (Fig 1), which influences the composition of the platform microbiota [45]. Kungsträdgården was designed by the artist Ulrik Samuelson as an underground cave garden (Fig 2), and the walls were deliberately not covered in concrete, as most stations belonging to the Stockholm metro system. Instead, they were left as bare granite. This further results in seeping groundwater on the rock walls with abundant speleothem formation and biofilm growth [46]. Although the bedrock is granite and the usual Si-based speleothem forming minerals were to be expected [44, 53], the speleothems contain only calcite [46].

The samples of the current study were taken from the north-facing wall, where an abundant fungal biofilm has developed on the granite wall covering an area of about 5 × 5 m^2^. The biofilm was sampled with stainless steel forceps washed in 70% alcohol. The sampling was conducted with gloved hands and measures were taken to minimize contamination. The samples were placed in 2 ml Eppendorf tubes and put on ice.

Because the sampling was carried out in a metro system, which is a public area, no specific permission and approval from authorities were required. During our study, no protected species were sampled.

### Fungal Strains, Morphology and Physiology

Samples of the collected material (ca. 5 mm^3^) were dispersed in 50 mL of distilled water. The suspension was then diluted 100×, 1000× and 10000×. A volume of 0.1 mL of the suspension was spread over the surface of 90 mm agar plates with a sterile bent glass rod ‘Trigalskii spathula’. Three types of media were used for isolation: 2% malt extract agar (MEA; Oxoid, Basingstoke, UK), MEA with 100 ppm of chloramphenicol, and dichloran-rose bengal agar (DRBC] [54]. In order to exclude growth of algae and cyanobacteria, the plates were incubated in the dark at 4, 10 and 17°C and inspected every week for a period of two months. Fungal colonies were transferred to new MEA plates and incubated at 17°C.

All isolates examined in this study were deposited in the Culture Collection of Fungi (CCF) at the Department of Botany, Charles University, Prague, Czech Republic; selected isolates were deposited in the Centraalbureau voor Schimmelcultures (CBS), Utrecht, The Netherlands. Herbarium specimens, i.e., dry cultures of newly described species, were deposited in the herbarium of the Mycological Department, National Museum (PRM) in Prague, Czech Republic and in the herbarium of the Swedish Museum of Natural History (S) in Stockholm, Sweden.

Axenic cultures were transferred onto nutrient media; MEA, potato-dextrose agar (PDA; Himedia, Mumbai, India) and potato-carrot agar (PCA) [55] sealed with laboratory film Parafilm M® (Bemis Company, Inc.). Micromorphology was observed and documented after 6‒8 wks of incubation with Melzer's reagent used as a mounting medium. All measurements were made in Melzer's reagent. Growth parameters at 10, 17 and 25°C were determined on MEA and measured at intervals of 2, 4 and 6 wks. The maximum growth temperature was determined on MEA by using incubation at 28, 30 and 32°C. Colour determination was performed according to the ISCC-NBS Centroid Colour charts [56]. Staining of heat-killed fungal hyphae with propidium iodide (Fluka) was used to observe nuclear numbers under fluorescence microscope. Microphotography was conducted with an Olympus BX-51 microscope with an Olympus DP72 camera and Nomarski contrast. Macromorphology of the colonies was documented with a Canon 500D camera or an Olympus SZ61stereo microscope with the camera. Photographs were processed with Helicon Focus, Creative Suit 6 Adobe Photoshop and CorelDraw Graphics Suite X4.

### DNA Extraction, Amplification and Sequencing of Environmental Samples

DNA was extracted from two environmental samples (F6 and F6a) using the CTAB method [57], with the difference that instead of grounding the material manually it was grounded in a TissueLyser LT (Qiagen) with two stainless steel beads at 5000 rpm for 2 min. About 0.1 g of biofilm material was used from each sample. DNA was purified with QIAquick® PCR-kit (Qiagen, Solna, Sweden/Hilden, Germany) following the instructions provided by the manufacturer. The extracted DNA was diluted in a 10-fold series up to 100-fold for use as PCR template.

Amplifications were carried out in a GeneAMP PCR System 9700 (Applied Biosystems, Singapore). The PCR protocols were as follows: 50 μl reaction volumes including 5 μl reaction buffer, 5 μl TMACL, 4 μl dNTP, 0.5 μl Paq5000 DNA polymerase (Agilent Technologies, Santa Clara, California) (5 U/μl), 0.7 μl of each primer (20 μM), 1.5 μl DMSO, 0.5 μl BSA 1%, 1 μl of DNA template and sterilized water up to 50 μl.

The nuclear large subunit ribosomal DNA (nuc28S rDNA) was amplified with primers LR0R (Vilgalys, unpublished: http://sites.biology.duke.edu/fungi/mycolab) and LR7 [58]. Amplification of partial nuclear small subunit ribosomal DNA (nuc18S rDNA) and the internal transcribed spacer (ITS1-5.8S-ITS2) operon (ITS rDNA) was conducted using primers nu-SSU-0817-5’ [59] and ITS4 [60]. Testing for the presence of Oomycota entailed oomycete-specific primer pairs for amplification of the ITS and cytochrome oxidase I (*coi*). For the ITS region, DC6 [61] and ITS4 were used. This primer pair targets a 1310 bp product in all Oomycota [62]. For amplification of *coi*, the forward primer OomCoxI-Levup was used in combination with the reverse primers Fm85mod or OomCoxI-Levlo [63].

The PCR profile for the nuc28S was: initial denaturation 95°C for 2 min followed by 35 cycles of (95°C, 25 s; 54°C, 25 s; 72°C, 1 min) and a final elongation step of 72°C for 5 min. The PCR profile for the nuc18S-ITS region was: initial denaturation 95°C for 2 min followed by 4 cycles of (95°C, 25 s; 56°C, 25 s; 72°C, 1 min and 15 s), 4 cycles (95°C, 25 s; 53°C, 25 s; 72°C, 1 min and 15 s), 32 cycles of (95°C, 25 s; 53°C, 25 s; 72°C, 1 min) and a final elongation step of 8 min. The PCR profile for the ITS region of the Oomycota was: initial denaturation 95°C for 2 min followed by 4 cycles of (95°C, 25 s; 56°C, 25 s; 72°C, 1 min and 15 s) 4 cycles (95°C, 25 s; 53°C, 25 s; 72°C, 1 min and 15 s) 32 cycles of (95°C, 25 s; 53°C, 25 s; 72°C, 1 min) and a final elongation step of 8 min. The PCR profile for *coi* of the Oomycota was: initial denaturation 95°C for 2 min followed by 35 cycles of (95°C, 25 s; 54°C, 25 s; 72°C, 30 s) and a final elongation step of 72°C for 5 min. PCR products were purified using Multiscreen PCR purification (Millipore, Billerica, MA, USA) according to the manufacturer's instructions.

Automated sequencing was carried out by Macrogen Sequencing Service (Amsterdam, the Netherlands). The nuc28S products were sequenced with the PCR primers and the nested primers LR5 [58] and LR3R (Vilgalys, unpublished: http://sites.biology.duke.edu/fungi/mycolab/primers.htm). The nuc18S-ITS region was sequenced with the PCR primers and the nested primers NS6 and NS7 [60]. The raw sequence data obtained from Macrogen were assembled, examined and edited using the Staden package v.2.0.0b9 [64].

### DNA Extraction, Amplification and Sequencing of Fungal Isolates

DNA was extracted from 3‒4 wks old colonies with ArchivePure DNA yeast and Gram2+ kit (5PRIME Inc., Gaithersburg, Maryland) with modifications described by Hubka et al. [65].

The nuc18S rDNA region was amplified with primers NS1 [60] and NS24 [66]; the fragment containing partial nuc18S, the entire ITS region, and partial nuc28S was amplified with primers V9G [67] and LR5 [58]. The largest subunit of RNA polymerase II (*rpb*1) was amplified and sequenced with primers RPB1-Afasc or RPB1-Af/6R1asc or 6R2asc [68, 69]. The DNA replication licensing factor (*mcm*7) was amplified and sequenced using primers Mcm7-709for and Mcm7-1348rev/Mcm7-1447rev [70]. Our various attempts to amplify β-tubulin and the second largest subunit of RNA polymerase II (*rpb*2) were unsuccessful.

The PCR mixture (25 μL total volume) contained 0.1 μL of MyTaq DNA polymerase (Bioline GmbH, Luckenwalde, Germany) (5 U/μl), 5 μL of MyTaq Reaction Buffer, 1μL of each primer (10 μM stock concentration), and 1μl (50 ng) of genomic DNA. PCR protocol was described by Hubka et al. [71]. PCR product purification: 2 μl of 3M sodium acetate and 60 μl of 96% cold ethanol were added to 20 μl of the PCR product. The mixture was vortexed, incubated for 20 min at 4°C, centrifuged for 30 min at 13200 rpm and 4°C. The supernatant was discarded and the pellet was dissolved in 175 μl of 70% cold ethanol. The mixture was centrifuged for 15 min at 13200 rpm and 4°C. The supernatant was discarded and the pellet was left for 10 min at room temperature and dissolved in 10 ml of deionized water.

Automated sequencing was performed at Macrogen Sequencing Service using both terminal primers and internal primers NS4 and NS5 [60] for nuc18S fragment and ITS4 [60] for the nuc18S-ITS-nuc28S fragment. The *rpb*1 segments A–D and *mcm7* were sequenced with the PCR primers. The raw sequence data were assembled, examined and edited using the BioEdit v.7.1.8 [72].

### Sequence Alignment

GenBank and EMBL (European Molecular Biology Laboratory database) accession numbers for ITS, nuc28S, nuc18S, *rpb*1, *rpb*2 and β-tubulin sequences determined for this study and other homologous sequences of members of the Chaetothyriales, Phaeomoniellales, Pyrenulales and Verrucariales (Chaetothyriomycetidae) retrieved from GenBank are listed in S1 Table. Majority of these taxa were published by [3, 7, 8, 17, 73–76].

We generated sequences of *mcm*7 for *Bradymyces alpinus* Hubka et al., *B*. *oncorhynchi* Hubka et al., the new genus described below and *Arthrocladium caudatum* Papendorf (summarized in S1 Table). However, this marker has not been sequenced for the majority of the Chaetothyriales, except members of the Cyphellophoraceae, and, therefore, it was not included in our datasets.

All sequences were manually aligned in BioEdit v.7.1.8. The nuc18S and nuc28S alignments were enhanced by utilizing the homologous 2D structure of *Saccharomyces cerevisiae* Meyen ex E.C. Hansen [77, 78] in order to improve the decisions on homologous characters and introduction of gaps. These procedures and alignment of the protein-coding genes were performed as described in [79]. Ambiguous regions and introns were delimited manually and excluded from the alignment. 2D structure models of the ITS1 and ITS2 previously obtained for all members of the Chaetothyriales [7] and the recently predicted models for *Bradymyces* were used to determine positions of homologous nucleotides in the ITS alignment and to improve the introduction of gaps.

The single-locus data sets were examined for topological incongruence among loci (ITS: 69 sequences/845 characters, β-tubulin exons 3−6: 14/528, nuc28S: 70/1222, nuc18S: 52/1750, *rpb*1 segments A−D: 35/1210, *rpb*2 segments 5−7: 14/1155). The ITS and β-tubulin loci were examined only for members of the Chaetothyriales. For each individual partition, 500 bootstrap replicates were generated with RAxML-HPC v.7.0.3 [80, 81] and compared visually for topological conflict among supported clades in phylogenetic trees. A conflict between two loci was assumed to occur when a clade appeared monophyletic with bootstrap support of ≥75% in one tree, but was not supported as monophyletic in another [82]. The conflict-free alignments were concatenated; the ITS alignment and two multi-locus alignments were subjected to subsequent phylogenetic analyses. The multiple sequence alignments are deposited in TreeBASE (Study no. 19251).

### Phylogenetic Analyses

Phylogenetic relationships of thirteen strains and two environmental samples of two unidentified black yeasts were resolved by three analyses based on the ITS sequences, combined sequences of ITS, nuc28S and β-tubulin of members of the Chaetothyriales and nuc18S, nuc28S, *rpb*1 and *rpb*2 sequences of the Chaetothyriomycetidae. We analysed the whole ITS rDNA barcode, the first two-thirds of the 5’ half of the nuc28S, the entire nuc18S, segments A−D of *rpb*1, segments 5−7 of *rpb*2, and exons 3−6 of β-tubulin. The first 120 nucleotides of the nuc18S, 65 of the nuc28S and 50 of the *rpb*2 alignments at the 5’-end were excluded from analysis because of the incompleteness of the majority of the available sequences. Two members of the Leotiomycetes, *Geoglossum nigritum* (Pers.) Cooke and *Trichoglossum hirsutum* (Pers.) Boud., two members of the Verrucariales, *Polyblastia viridescens* Zschacke and *Verrucaria rupestris* Schrad., and two species of *Strelitziana* Arzanlou & Crous were used to root the individual trees.

The combined datasets were partitioned into several subsets of nucleotide sites, i.e., ITS, nuc18S, nuc28S, *rpb*1, *rpb*2 and coding and non-coding regions of β-tubulin. Maximum likelihood (ML) and Bayesian inference (BI) analyses were used to estimate phylogenetic relationships. ML analysis was performed with RAxML-HPC v.7.0.3 with a GTRCAT model of evolution. Nodal support was determined by non-parametric bootstrapping (BS) with 1 000 replicates. BI analysis was performed in a likelihood framework as implemented in MrBayes v 3.0b4 to reconstruct phylogenetic trees [83]. For the BI approach, MrModeltest2 v.2.3 [84] was used to infer the most suitable substitution model; the model was selected according to an Akaike information criterion for seven partitions for which we assumed rate heterogeneity. For the ITS, nuc18S, nuc28S, rpb1 and rpb2 data sets, we used for each partition GTR+I+G substitution model, and SYM+G model for the ITS of *Bradymyces* spp. For the coding regions of β-tubulin we used GTR+G model and HKY+I+G for the non-coding regions. Two Bayesian searches were performed using the default parameters. Analyses were run for 10 million generations, with trees sampled every 1 000 generations. Tracer v.1.6.0. [85] was used to confirm convergence of trees and burn-in. The first 50 000 trees, which represented the burn-in phase of the analysis, were discarded. The remaining trees were used for calculating posterior probabilities (PP) of recovered branches [86].

### Prediction of 2D Structure Models of ITS1 and ITS2 of *Bradymyces*

Consensus 2D structure models for the ITS1 and ITS2 of members of the Chaetothyriales previously obtained by Réblová et al. [7] were used to construct 2D models for *Bradymyces* [49]. These models were built using the PPfold program v.3.0 [87], which uses an explicit evolutionary model and a probabilistic model of structures, and relies on multiple sequence alignment of related RNA sequences. The obtained consensus models were further improved using Mfold program [88] and then adjusted manually if necessary, based on a comparison of homologous positions in the multiple sequence alignment. The predicted 2D RNA structures were obtained in a dot-bracket notation and were visualized and drawn using VARNA: Visualization Applet for RNA program [89]. The final 2D models were processed with CorelDRAW Graphics Suite X4.

Three types of substitutions were identified in the aligned ITS sequences of *Bradymyces* spp. The compensatory base changes (CBCs) occur when both nucleotides of a paired site with a canonical pair mutate, i.e., G = C ↔ C = G, A-U or U-A. The hemi-compensatory base changes (hCBCs), also called one-sided substitutions, inflict the change of a canonical base pair to a ‘wobble’ base pair, i.e., G = C → G/U. The non-compensatory base changes (non-CBC) are the third type of substitutions that involve the replacement of a canonical pair or a wobble pair with any non-canonical pair. While the CBCs and hCBCs are responsible for maintaining the arrangement of base pairs in the RNA transcript and preserving the structure of the RNA helices, non-CBCs lead to the disruption of the stem structure [90]. Once all existing substitutions were identified among species and unnamed monophyletic clades of *Bradymyces*, they were mapped onto the predicted 2D structures of ITS1 and ITS2 of *B*. *oncorhynchi*, the generic type.

### Nomenclature

The electronic version of this article in Portable Document Format (PDF) in a work with an ISSN or ISBN will represent a published work according to the International Code of Nomenclature for algae, fungi, and plants, and hence the new names contained in the electronic publication of a PLOS article are effectively published under that Code from the electronic edition alone, so there is no longer any need to provide printed copies.

In addition, new names contained in this work have been submitted to MycoBank from where they will be made available to the Global Names Index. The unique MycoBank number can be resolved and the associated information viewed through any standard web browser by appending the MycoBank number contained in this publication to the prefix http://www.mycobank.org/MB/. The online version of this work is archived and available from the following digital repositories: [PubMed Central, LOCKSS].

## Results

### Environmental Samples

Amplification products using the oomycete-specific primer pairs for ITS and *coi* were consistently blank. Amplification of the nuc28S and nuc18S-ITS regions resulted in single bands of expected length for the 1:100 dilution templates. Amplification of the undiluted and the 1:10 dilution templates did not result in any product. This is most likely due to that these templates had high concentrations of PCR inhibitors.

The raw sequencing reads were ‘clean’, i.e., without double peaks and could be readily assembled. The nuc28S and the nuc18S-ITS sequences of two environmental samples F6 and F6a were identical. Based on the BLAST searches (21/04/2015), both nuc28S sequences showed 99% similarity (1322/1329 bp) with *Bradymyces alpinus* CCFEE 5493 (GU250396). Their nuc18S-ITS region showed 99% similarity (959/963 bp) with *B*. *oncorhynchi* (HG426064) and (962/965 bp) with *B*. *alpinus* CCFEE 5493 (GU250354). The sequences of environmental samples were subjected to two phylogenetic analyses.

### Spectrum of Fungi Isolated from the Speleothem Forming Biofilm

Six isolates of slow-growing melanised fungi described in our study as *Bradymyces graniticola* were first observed and isolated after 4‒6 wks of incubation on MEA at 10°C (dilution 1000× or 10000×). Two strains of another melanised morphotype introduced below as *Bacillicladium lobatum* were isolated after 2 and 3 wks of incubation on MEA or DRBC at 17°C (dilution 100× and 1000×). Several other strains of filamentous fungi belonging to various lineages of Ascomycota and Basidiomycota were successfully isolated in axenic culture and sequenced for ITS rDNA. They include *Cladosporium* spp., *Acremonium nepalense* W. Gams, *Penicillium expansum* Link, and the basidiomycete yeast-like species *Trichosporon* cf. *akiyoshidainum* Sugita et al.

### Phylogenetic Results

**Fig 3:**
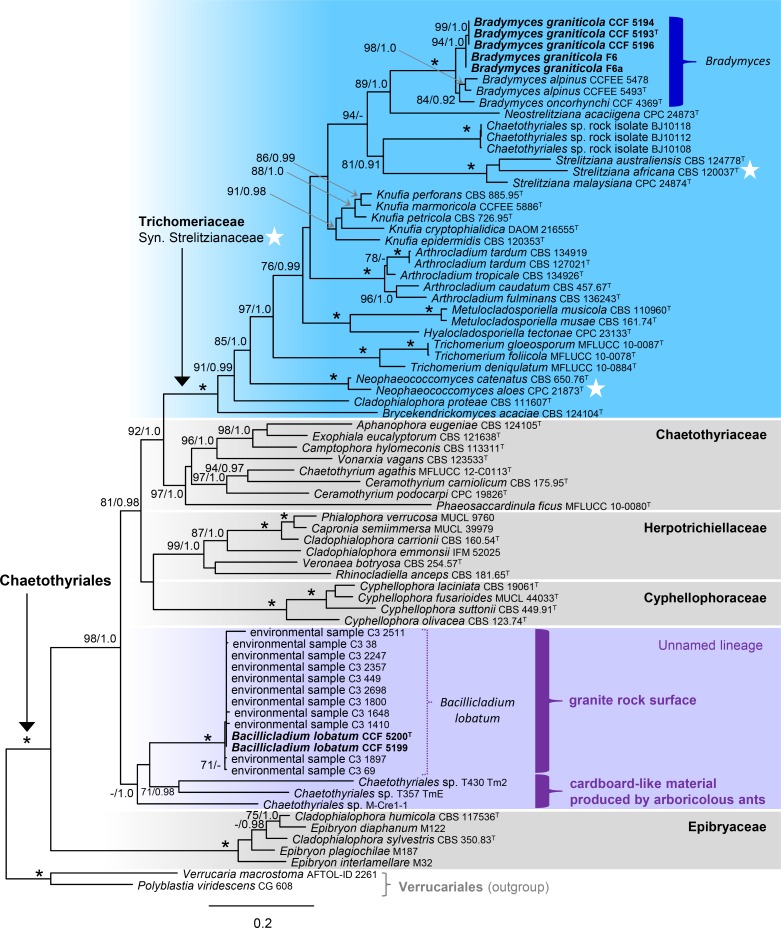
Phylogenetic analysis of the nucITS-nuc28S-β-tubulin sequences of the Chaetothyriales. Branch support in nodes ≥ 70% maximum likelihood bootstrap support (ML BS) and ≥ 0.90 Bayesian posterior probability (PP) are indicated above or below branches. The asterisk (*) indicates nodes with 100% ML BS/1.0 PP. Taxa written in bold represent taxonomic novelties. ^T^ = ex-type.

In the first analysis, 76 combined ITS, nuc28S and β-tubulin sequences were assessed for 55 species in five families of the Chaetothyriales. The alignment had 1300 distinct alignment patterns (ML analysis). In the ML tree shown in Fig 3, the Chaetothyriales (100 ML BS/1.0 PP) are resolved with six nested clades. Five of these monophyletic clades represent the families Chaetothyriaceae (97/1.0), Cyphellophoraceae (100/1.0), Epibryaceae (100/1.0), Herpotrichiellaceae (99/1.0) and Trichomeriaceae (100/1.0). The sixth unnamed lineage is basal to a well-supported clade (81/0.98) containing all families except the Epibryaceae. It includes two strains CCF 5199 and CCF 5200 of the unidentified black yeast isolated from the speleothem biofilm (Sweden) and eleven environmental samples from subaerial biofilm on granite (Spain, S1 Table). They form a strongly supported monophyletic clade (100/1.0), which is described as the new monotypic genus *Bacillicladium* (*Ba*.) in our study. A sister relationship of *Bacillicladium* and three strains of chaetothyrialean fungi, T357 TmE, T430 Tm2 and M-Cre1-1, isolated from nests of tropical ants, is weakly supported in ML analysis while strongly supported in BI (67/1.0). Three isolates of the second unknown black yeast CCF 5193, CCF 5194 and CCF 5196 and two environmental biofilm samples F6 and F6a are conspecific based on molecular data and are grouped together with *Bradymyces alpinus* and *B*. *oncorhynchi* in the Trichomeriaceae (100/1.0). They represent a new *Bradymyces* species described below. *Neophaeococcomyces* Crous & M.J. Wingf., type species *N*. *aloes* (Crous & M.J. Wingf.) Crous & M.J. Wingf., and *Strelitziana* Arzanlou & Crous, type species *S*. *africana* Arzanlou & Crous, the only members of the *Strelitzianaceae*, are here shown to be distantly related to each other but nested within the multigeneric family Trichomeriaceae.

**Fig 4:**
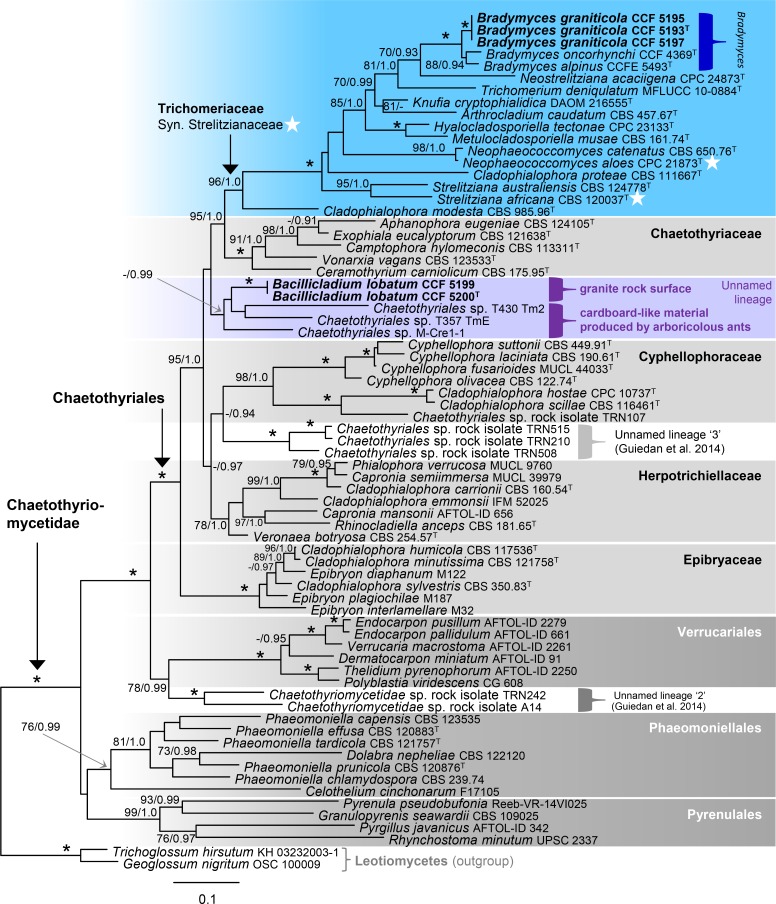
Phylogenetic analysis of the combined nuc18S-nuc28S-*rpb*1-*rpb*2 sequences of the Chaetothyriomycetidae. Details as in Fig 3.

The second data set consisted of 71 combined nuc18S, nuc28S, *rpb*1 and *rpb*2 sequences of members of the Chaetothyriomycetidae. The alignment had 2402 distinct alignment patterns. The ML tree is shown in Fig 4. The Chaetothyriomycetidae comprise four strongly to well-supported monophyletic clades, i.e., the Chaetothyriales (100/1.0), Phaeomoniellales (76/0.99), Pyrenulales (99/1.0) and Verrucariales (100/1.0). The unnamed lineage in the Chaetothyriales comprising two strains of *Bacillicladium lobatum*, CCF 5199 and CCF 5200, and three strains of ant-associated fungi, T357 TmE, T430 Tm2 and M-Cre1-1, was not statistically supported in ML analysis (below 50%), but obtained high support in BI (0.99). This lineage has slightly different topology than in the 3-gene analysis; it is shown basal to a robust clade containing the Chaetothyriaceae and Trichomeriaceae. The genus *Bradymyces* including *B*. *alpinus*, *B*. *oncorhynchi* and the new species *B*. *graniticola* forms a monophyletic clade (100/1.0) in the Trichomeriaceae. The placement of type species of *Neophaeococcomyces* and *Strelitziana* is congruent in topologies of the three- and four-gene phylogenies. Both genera are grouped in the Trichomeriaceae.

**Fig 5:**
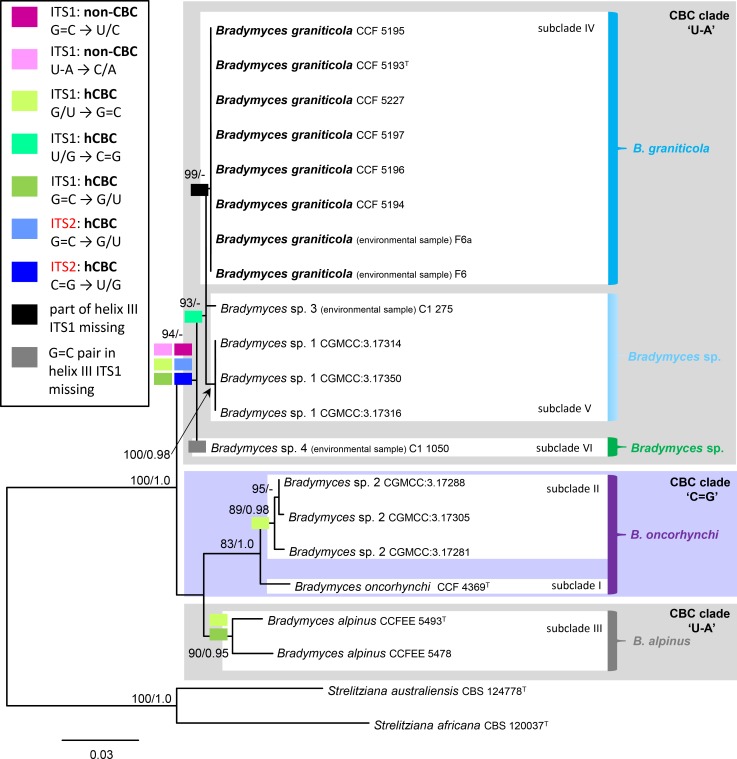
Phylogenetic analysis of the ITS rDNA sequences of *Bradymyces* spp. Groups of taxa with no CBC among them are labelled as CBC clades indicated by colour blocks. The non-CBCs and hCBCs substitutions identified in ITS1 and ITS2 2D structure are colour-coded as shown in the legend in the frame; respective internodes are labelled with them. The first base pair in the legend always refers to *B*. *oncorhynchi*. Details as in Fig 3.

The ITS data set consisted of 19 sequences of *Bradymyces* spp; six isolates and two environmental samples of the new species, two strains of *B*. *alpinus*, a strain of *B*. *oncorhynchi* and other rock isolates and environmental samples. The alignment had 158 distinct alignment patterns. The ML tree is shown in Fig 5. Based on a single double-sided substitution (CBC: C = G → U-A) encountered in the highly conserved helix II ITS2 secondary structure among all analysed sequences of *Bradymyces* spp. (for details see below), the resolved clades were labelled accordingly, i.e., taxa without any CBC among them were designated a CBC clade. The CBC clade including *B*. *oncorhynchi* and *Bradymyces* sp. 2, and the second CBC clade with *B*. *alpinus*, *B*. *graniticola* and *Bradymyces* sp. 1, 3, 4. The latter CBC clade was shown paraphyletic. The clades are divided into six subclades that are further characterised by hCBCs and non-CBCs events in the ITS1 and ITS2 secondary structure that are mapped onto the phylogram (Fig 5) and discussed below. Although the topology of ML tree is largely identical to the BI tree, the internodes are well- or strongly supported in the ML analysis while in the BI the statistical supports of several clades were lower than 90%, and are not shown at the nodes.

### Consensus 2D Structure of ITS1 and ITS2 of *Bradymyces*

**Fig 6:**
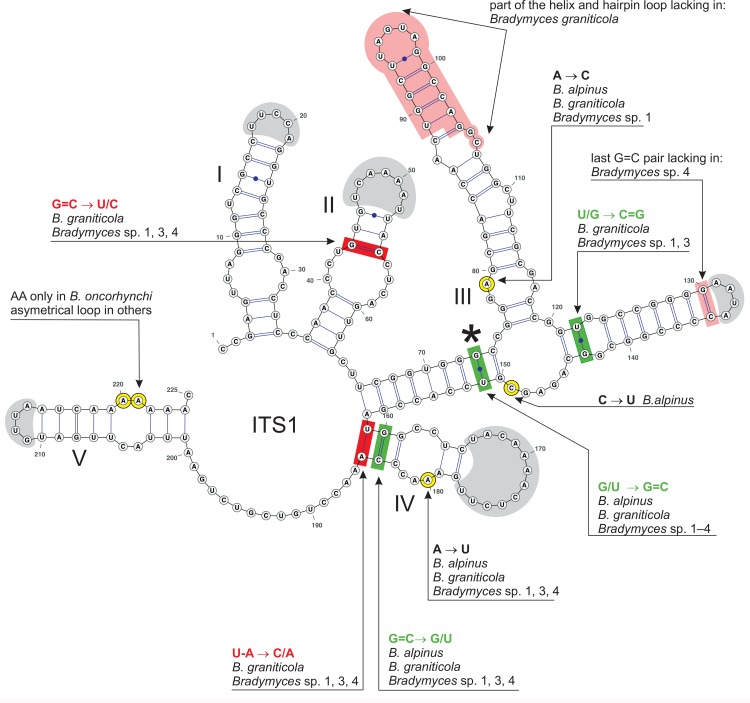
ITS1 secondary structure of *Bradymyces oncorhynchi* (HG426062). ITS1 helices are numbered I–V. All substitutions recorded among members of *Bradymyces* are mapped on the 2D model. The asterisk (*) marks the only hCBC between *B*. *oncorhynchi* and *Bradymyces* sp. 2. Legend to symbols and colours as in Fig 7.

**Fig 7:**
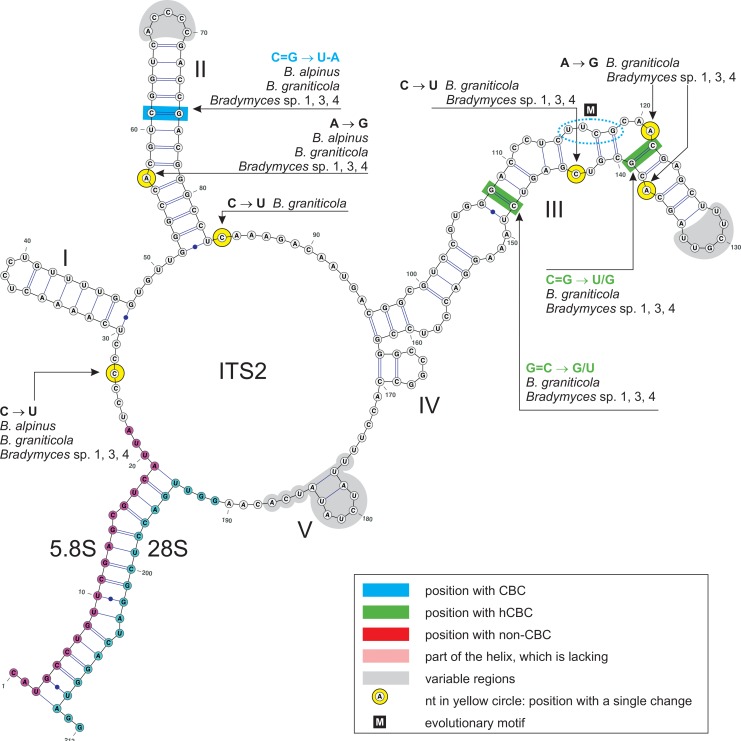
ITS2 secondary structure of *Bradymyces oncorhynchi* (HG426062) and 5.8S-28S rRNA gene hybridization (proximal stem region). ITS2 helices are numbered I−V. All substitutions recorded among members of *Bradymyces* are mapped on the 2D model. Identified substitutions are colour-coded: CBC (blue), hCBC (green) and non-CBC (red); single substitution (yellow). Parts of hairpin loops, junctions and a helix highlighted with grey colour represent regions with a variable number of nucleotides or sequence variation.

The 2D predicted structures of ITS1 (Fig 6) and ITS2 (Fig 7) are modelled for the type species *B*. *oncorhynchi*. The consensus 2D structure of ITS1 consists of five helices I−V separated by a single nucleotide or junction regions. The helices I, II, IV and V exhibit a symmetrical internal loop and a hairpin loop of a similar length with a slight sequence variation among terminal clades distinguished in *Bradymyces* (Fig 5). Helix III is the longest, folded into a three-way junction (3WJ) with two short asymmetrical and one symmetrical internal loop in the right arm. The hairpin loop and the upper part of the right arm of helix III are lacking in all six isolates and two environmental samples of *B*. *graniticola* (Fig 6). No CBC was identified among analysed sequences. Only three hCBCs occur in ITS1, two on helix III and one on helix IV. Helices II and IV show each one non-CBC.

The consensus 2D structure of ITS2 is folded into the common core structure typical for Eukaryota, i.e., a ring structure with four main helices I−IV, of which helix II (32 nt) and helix III (67 nt) are the longest and highly conserved. A fifth short and highly variable helix was positioned on the ring structure near the 3´-end in all species of *Bradymyces*. The only CBC identified in ITS2 occurs on the ninth base pair of helix II and entails a double-sided substitution, i.e., when the C = G pair in *B*. *oncorhynchi* and *Bradymyces* sp. 2 is mutated to a U-A pair in *B*. *alpinus*, *B*. *graniticola* and *Bradymyces* sp. 1, 3, 4. Two hCBCs were identified on helix III, the non-CBC was not encountered. The pyrimidine-pyrimidine mismatch in helix II was not observed, which is in agreement with our previous observations of ITS2 2D of members of the Chaetothyriales [7].

### Taxonomy

Trichomeriaceae Chomnunti & K.D. Hyde, Fungal Diversity 56: 66. 2012.

Syn.: Strelitzianaceae Crous & M.J. Wingf., Persoonia 35: 287. 2015.

Comments. The recently described *Strelitzianaceae* [10] in the order Chaetothyriales includes two genera, *Strelitziana* and *Neophaeococcomyces*. The latter genus was introduced for two *Phaeococcomyces* De Hoog species, while the generic type *P*. *nigricans* (M.A. Rich & A.M. Stern) de Hoog is positioned in the Lichenostigmatales, Dothideomycetes [91]. The introduction of the new family was inadvertently distorted by the inclusion of only members of these two genera in the absence of sequences from other representatives of the Chaetothyriales. In fact, *Strelitziana* and *Neophaeococcomyces* represent only a small part of the previously recognized Trichomeriaceae. In our multilocus phylogeny, these two genera grouped on separate branches among other members of the multigeneric family Trichomeriaceae (Figs 3 and 4).

***Bacillicladium*** Hubka, Réblová & Thureborn, gen. nov.

[urn:lsid:indexfungorum.org:names:816983]

*Conidiomata* absent. Three types of growth habit are present, hyphal, meristematic and yeast-like. *Vegetative hyphae* smooth, septate, moniliform, almost hyaline to brown, unbranched or sparsely branched, with uni- or bicellular chlamydospore-like hyphal swellings, intercalary, brown, thick-walled, ellipsoidal, obovate, subglobose or globose. The colony surface sometimes covered by a felt of erect, unbranched moniliform hyphae growing through the blastic proliferation of the apical cell; hyphae of variable length divided into two different types consisting either of shorter and thicker aseptate or ostensibly uniseptate cells (septum is incomplete and probably represents only cell wall reinforcement) or long and thinner cells appearing multiseptate. Uni- or multicellular bodies are formed in culture, single or in chains. *Meristematic parenchyma-like structure* formed in the centre of the colony, releases brown irregularly shaped uni- or multicellular elements with roughened walls due to incrustations on their surface. *Yeast-like cells* single or in short chains, aseptate or one-septate, hyaline to mid-brown, ellipsoidal, obovate, subglobose or globose, budding at the narrow end. *Endoconia* absent. *Sexual morph* unknown.

Etymology. *Bacillus* (L), rod; referring to erect simple hyphae at the surface of the colony consisting of elongated rod-shaped elements. *Cladium* (Gk) from Greek klados = branch.

Type species. *Bacillicladium lobatum* Hubka, Réblová & Thureborn

**Fig 8:**
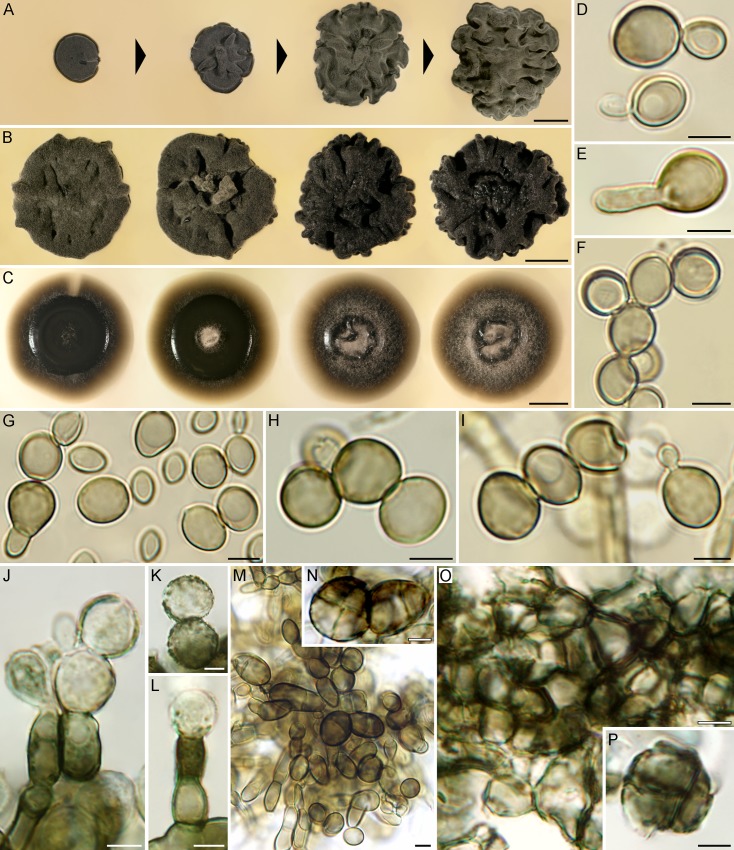
Macromorphology and micromorphology of *Bacillicladium lobatum*. (A) Morphogenesis of colony growing on MEA at 25°C in 9 d, 2, 4 and 6 wks (left to right). (B, C) Phenotypic variability of 6-week-old colonies at 25°C on PDA (B) and PCA (C). (D‒I) Yeast-like state, budding (D, I), germinating by hyphae (E, G) or forming short chains (F‒I). (J‒L) Fungal elements from the inner parts of the colony with incrustations on their surface, occasionally proliferating. (M, N) Uni- or multicellular bodies, single or in chains. (O) Meristematic parenchyma-like structures formed in the inner parts of the colony (MEA). (P) Multicellular element released from the parenchymatous structure with roughened wall. Bar = 5 mm (A‒C), 5 μm (D‒P).

**Fig 9:**
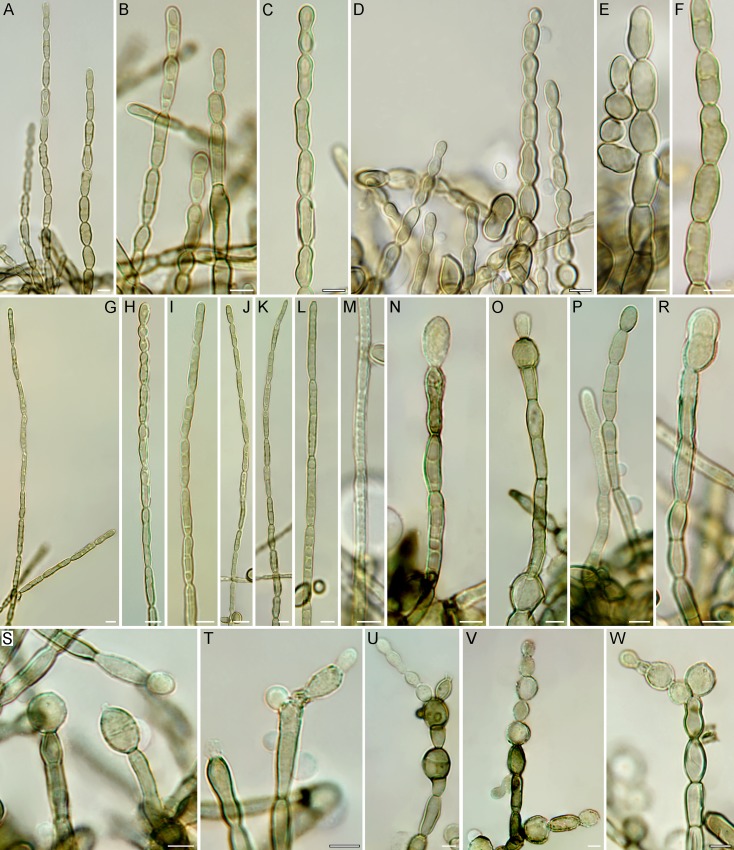
Hyphal state of *Bacillicladium lobatum*. (A–M) Unbranched hyphae growing through blastic proliferation and covering surface of colonies on MEA and PDA: (A–F) hyphae of the first type, aseptate or ostensibly uniseptate, commonly constricted in the middle part; (G–M) hyphae of the second type with numerous cell wall reinforcements and multiseptate appearance. (N–W) Unbranched or sparsely branched vegetative hyphae from the deeper parts of the colonies, occasionally terminated by a swollen globose to ellipsoidal cell (N, O, R–T), sometimes with signs of budding (O, S, T), occasionally with uni- or bicellular chlamydospore-like hyphal swellings intercalary in position (U, V). Bar = 5 μm.

**Fig 10:**
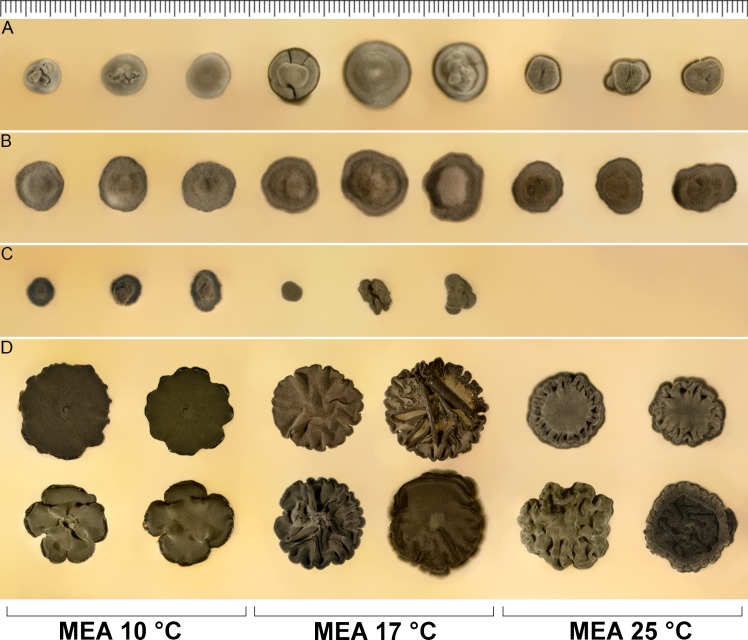
Morphology and variability of colonies and growth parameters of *Bradymyces* spp. and *Bacillicladium lobatum* after 6 wks of cultivation on MEA at three different temperatures. (A) *B*. *graniticola*. (B) *B*. *oncorhynchi*. (C) *B*. *alpinus*. (D) *Ba*. *lobatum*. One ‘tick’ on the ruler in the upper part of the figure corresponds to 1 mm.

***Bacillicladium lobatum*** Hubka, Réblová & Thureborn, sp. nov. (Figs 8A–8P, 9A–9W and 10D).

[urn:lsid:indexfungorum.org:names:816984]

*Colonies* slow-growing, on MEA at 25°C in 42 d attaining 14–23 mm diam (3–11 mm in 14 d), Grayish Olive (ISCC-NBS Colour No. 109) to Dark Grayish Olive (No. 111), velvety, raised, cerebriform, margin lobate, reverse black. Colonies on PDA at 25°C in 42 d attaining 10–18 mm diam (2–7 mm in 14 d), Dark Grayish Olive (No. 111), velvety to crusty, raised, cerebriform, margin lobed, reverse brown-black to black. Colonies on PCA at 25°C in 42 d attaining 13–16 mm diam (7–10 mm in 14 d), Olive Black (Nos. 114) in the center, Moderate Olive (No. 107) in the marginal part, moist, covered by sparse aerial mycelium, flat with a broad zone of submerged growth, margin entire, reverse brown-black to black. Colonies at 17°C showing similar growth parameters and slight morphological differences; colonies on MEA and PDA are less folded, colonies on PCA have a more moist appearance with less abundant aerial mycelium on the surface. Colonies on MEA at 10°C in 42 d attaining 15–19 mm diam (1–5 mm in 14 d), velvety, flat or with undulated surface, the margin moist undulate or coarsely lobed. Colonies on MEA at 28°C in 14 d attaining 4–7 mm diam, no growth observed on MEA at 30°C after 14 d.

*Vegetative hyphae* smooth, septate, almost hyaline, pale brown to mid-brown, 2.5–4 μm wide, becoming constricted at the septum, moniliform, definitely mid-brown to brown, (3.5–)4–6(–7) μm wide, unbranched or sparsely branched, occasionally terminated by a swollen globose to ellipsoidal cell, 8–11 × 6–7.5(–9.5) μm, which frequently buds at the apex. Uni- or bicellular chlamydospore-like hyphal swellings intercalary in position, 7–12 μm diam. The surface of the colony on MEA and PDA covered by a felt of erect, unbranched moniliform hyphae growing through blastic proliferation of the apical cell; hyphae of two types: hyphae of the first type up to 200 um long, composed of aseptate cells which may appear to be uniseptate, 8–12 × 3–5 μm, commonly constricted in the middle where always a single nucleus is located (fluorescence microscope); hyphae of the second type up to 700 um long, composed of longer cells with numerous cell wall reinforcements giving them multiseptate appearance, 10.5–25 × 2–3 μm, with a single nucleus located in variable position, most commonly in the peripheral part of each cell. Uni- or multicellular (up to 4 cells) bodies formed in culture, mostly single or in short chains, (8.5–)10–20.5 × 6.5–13.5 μm, mid-brown to brown, thick-walled, ellipsoidal, obovate, subglobose or globose. *Meristematic parenchyma-like structures* formed in the inner parts of the colony on MEA and PDA alternating with tightly attached hyphae. Uni- or multicellular elements released from the parenchymatous structure have roughened walls due to incrustations on their surface. *Yeast-like cells* single or in short chains, (4.5–)5–9 × 3–7(–8) μm, non-septate, rarely one-septate, hyaline when young, becoming mid-brown to brown, ellipsoidal, obovate, subglobose or globose, budding at the narrow end. *Endoconia* absent. *Sexual morph* unknown.

Holotype. Sweden, Stockholm, Kungsträdgårdens metro station, bare granite walls, 15 July 2015, leg. O. Thureborn, isol. Sept. 2015 *V*. *Hubka* S1K1 (PRM 935094 (PRM), holotype; PRM 935211−935213 (PRM), F286603 (S), F286606 (S), F286608 (S), F286610 (S), isotypes; culture ex-type CCF 5200 = CBS 141179).

Etymology. *Lobatus* (L), lobed; referring to the shape of the colony margin.

Additional specimen examined. Sweden, Stockholm, Kungsträdgårdens metro station, bare granite walls, 15 July 2015, leg. O. Thureborn, isol. Sept. 2015 *V*. *Hubka* S2K1, culture CCF 5199 = CBS 141180.

Comments. *Bacillicladium lobatum* exhibits three different growth habits *in vitro* whose abundance is dependent on cultivation medium, temperature and age of colony. The yeast-like phase is dominant at early stages of colony development on all media. It is subsequently replaced on MEA and PDA from the central part of the colony by a filamentous and later meristematic phase while the yeast-like phase becomes restricted to marginal parts and remains present only to a limited extent in the central part of the colony. In contrast, on PCA, the yeast-like phase remains dominant even in old colonies. The colony growth on MEA at 10, 17 and 25°C is shown in Fig 10.

**Fig 11:**
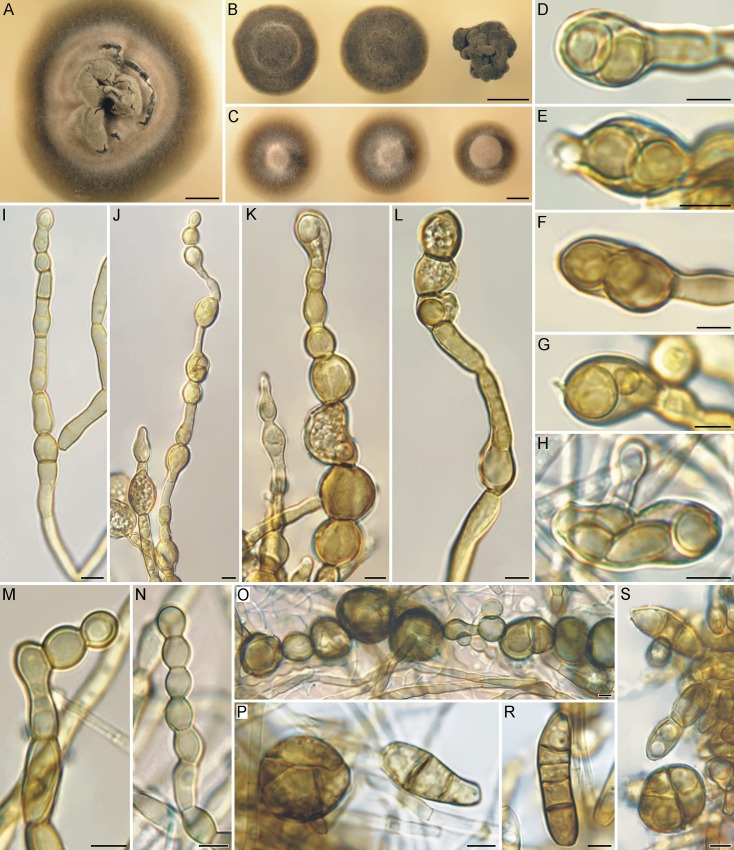
Macromorphology and micromorphology of *Bradymyces graniticola*. (A) A 16-week-old colony on MEA at 17°C. (B) Phenotypic variability of 6-week-old colonies on PDA at 25°C. (C) Phenotypic variability of 6-week-old colonies on PCA at 25°C. (D–H) Endoconidia. (I–N) Moniliform hyphae proliferating at the apex. (O) Chlamydospore-like hyphal swellings intercalary in position. (P–S) Multicellular bodies released from meristematic part of colony. Bar = 5 mm (A‒C), 5 μm (D‒S).

***Bradymyces graniticola*** Hubka, Réblová & Thureborn, sp. nov. (Figs 10A and 11A–11S)

[urn:lsid:indexfungorum.org:names:816985]

*Colonies* slow-growing, on MEA at 17°C in 42 d attaining 9–16 mm diam (1–5 mm in 14 d), Light Greenish Gray (ISCC-NBS Colour No. 154) to Grayish Yellow Green (No. 122), velvety, raised, hemispherical, less commonly cerebriform or with cracked surface, margin entire, reverse black. Colonies on PDA at 17°C in 42 d attaining 8–20 mm diam (1–5 mm in 14 d), Grayish Olive (No. 110) to Olive Gray (No. 113), velvety, raised, umbonate or cerebriform, margin entire, reverse black. Colonies on PCA at 17°C in 42 d attaining 8–20 mm diam (2–6 mm in 14 d), Yellowish Gray (No. 93) in the center, Moderate Olive (No. 107) in the margin parts, woolly, centrally raised with a broad zone of submerged growth, margin filiform, reverse brown-black to black. Colonies on MEA at 25°C showing similar morphology to MEA at 17°C and slower growth parameters, attaining 4–10 mm diam in 42 d (1–4 mm in 14 d). Colonies on MEA at 10°C showed similar morphology and growth parameters comparable to 25°C, attaining 5–9 mm diam in 42 d (1–4 mm in 14 d). No growth observed on MEA at 28°C after 14 d.

*Vegetative hyphae* smooth, septate, yellowish-brown to mid-brown, 1.5–4 μm wide, becoming constricted at the septum, moniliform, definitely mid-brown to brown, (3.5–)4–7 μm wide, unbranched or sparsely perpendicularly branched. Globose, ellipsoidal or irregularly-shaped, brown to dark brown, uni- or multi-cellular (up to 4 cells) chlamydospore-like hyphal swellings developing in intercalary and/or terminal positions or single, (8.5–)10–19(–24) μm diam. Blastic proliferation commonly present at the ends of moniliform hyphae, terminal cells sometimes larger than the subterminal cells, globose or elongated, often proliferating with umbonate apex due to budding, 6.5–14.5 μm long, 6–9.5 μm wide. *Endoconidia* unicellular, globose, subglobose or ellipsoidal, light brown, mostly 1–3 in number but up to 5, (3–)4.5–9 × (3–)3.5–5.5(–8) μm, parent cells mostly ellipsoidal, barrel-shaped, elongated, less commonly globose, terminal or intercalary, 9.5–20 × 6–18 μm. *Sexual morph* unknown.

Holotype. Sweden, Stockholm, Kungsträdgårdens metro station, bare granite walls, 15 July 2015, leg. O. Thureborn, isol. Sept. 2015 *V*. *Hubka* S10K1a (PRM 935092 (PRM), holotype; PRM 935093 (PRM), PRM 935214−935216 (PRM), F286611 (S), isotypes; culture ex-type CCF 5193 = CBS 140773).

Etymology. *Granites* (L), granite; *colo* (L), inhabit; referring to the occurrence of the fungus on the granite walls.

Additional specimens examined. Sweden, Stockholm, Kungsträdgårdens metro station, bare granite walls, 15 July 2015, leg. O. Thureborn, isol. Sept. 2015 *V*. *Hubka* S7K1a, culture CCF 5194 = CBS 140774; Ibid. isol. Sept. 2015 *V*. *Hubka* S8K1a, culture CCF 5196; Ibid. isol. Sept. 2015 *V*. *Hubka* S9K1a, culture CCF 5197; Ibid. isol. Sept. 2015 *V*. *Hubka* S9K1b, culture CCF 5227; Ibid. isol. Sept. 2015 *V*. *Hubka* S9K1c, culture CCF 5195.

**Fig 12:**
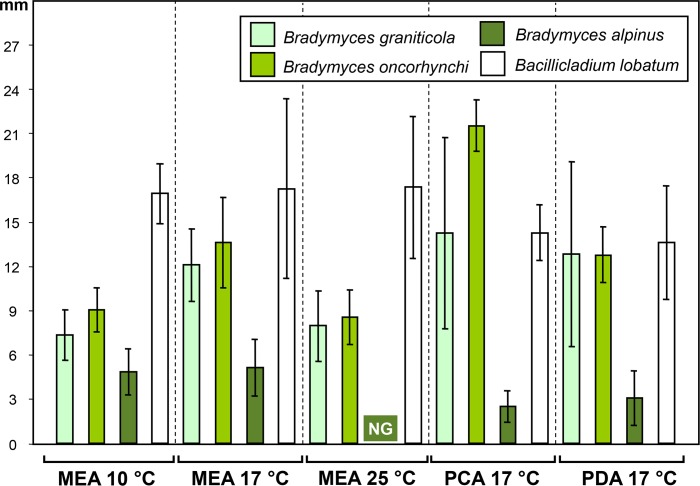
Colony diameters of *Bradymyces* spp. and *Bacillicladium lobatum* after 6 wks of cultivation on different media and temperatures. Isolates included: *B*. *graniticola* (CCF 5193−5197, CCF 5227); *B*. *oncorhynchi* (CCF 4369), *B*. *alpinus* (CCFEE 5493), *Ba*. *lobatum* (CCF 5199, CCF 5200). Each point represent mean of at least nine values per isolate; error bars represent standard deviations.

Comments. *Bradymyces graniticola* is strongly reminiscent of *B*. *oncorhynchi* in micro- and macromorphological characteristics, although minor differences can be found in the macromorphology of colonies, which are darker brown in *B*. *oncorhynchi* on MEA (Fig 10), and growth parameters on MEA at 10°C and PCA at 17°C (Fig 12). Both species are also characterized by inability to grow at 28°C. *Bradymyces alpinus* can be easily distinguished from both species by the inability to grow at 25°C and overall slower growth at 10°C as well as 17°C (Figs 10 and 12). Their distinction based on DNA sequence data (Figs 3–5) is strongly supported by ML analysis. *Bradymyces alpinus*, *B*. *oncorhynchi*, the novel species *B*. *graniticola* and several other *Bradymyces* spp. isolates and environmental samples are further distinguished at the RNA structural level (see Discussion).

## Discussion

### The Biofilm Microbiota

Biofilm is defined as „a microbial community attached to a solid surface composed of cells organised as microcolonies embedded in an organic polymer matrix of microbial origin” [92]. The development of the multispecies biofilm is a long process involving different successive steps and colonization of various microorganisms. A biofilm involves numerous microenvironments, in which particular microorganisms have to adapt based on availability of nutrients, light, water and oxygen.

**Fig 13:**
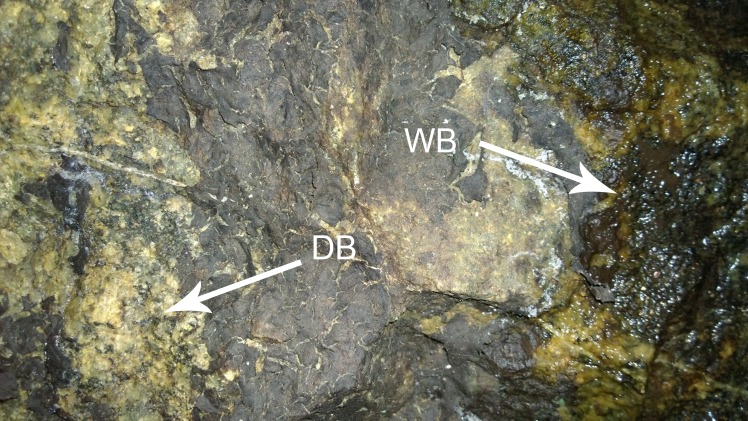
Biofilm on a granite wall. Part of the wall with both dry and wet areas covered by biofilm; DB = dry biofilm, WB = wet biofilm. Bar = 5 cm.

**Fig 14:**
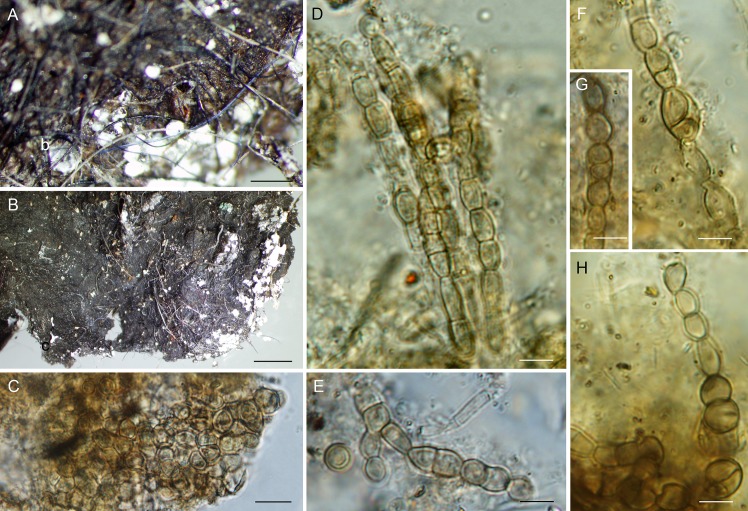
Biofilm containing fungal hyphae. (A) Removed biofilm containing fungal hyphae; note the tissue-like texture with protruding hyphae and white mineral precipitates. (B) Biofilm showing the fashion of fungal hyphae producing the tissue-like texture of the mat. (C) Fragment of meristematic tissue composed of isodiametric and angular cells. (D, E, H) Fragments of melanised monilioid hyphae. (F, G) Hyphae with endoconidia. Bar = 100 μm (A), 200 μm (B), 10 μm (C), 5 μm (D−H).

The granite wall in the Kungsträdgården metro station is exposed to unusual conditions causing biofilm growth to adapt to high humidity, but also to draught. The wall is subject to abundant seepage of groundwater conducted through a small pipe from the bedrock. The precipitation of mineral phases (calcium- and magnesium carbonates) makes the biofilm even more complex in nature. On parts of the wall which are wet, the biofilm appears smooth and green-brownish, while at the dried parts it looks like a dark brown crust (Fig 13). The biofilm at this site is unusually solid and tissue-like. The dense network of fungal hyphae forms almost a blanket-like mat (Fig 14A and 14B). Microscopic preparations of the native biofilm revealed melanised fungal hyphae or hyphal fragments with or without endoconidia and parts of pseudoparenchymatic tissue consisting of dark brown, thick-walled, almost isodiametric to angular cells (Fig 14C–14H). Constant illumination with artificial light favours the presence of a ‘Lampenflora’ of cyanobacteria, diatoms and the bryophyte *Eucladium verticillatum* (Brid.) Bruch & Schimp., which in turn support the presence of other heterotrophic organisms like Fungi, Collembola, Crustacea, Nematoda, Annelida, and the first reported habitat for the spider *Lessertia dentichelis* Simon in Sweden [46, 93].

The microorganisms appear to play an important ecological role as they exist and facilitate specialized niches on the granite walls. Diatoms, for instance, thrive in ‛diatom ooze’, produced by diatom mediated dissolution of calcium carbonate speleothems [45]. Fungal hyphae are commonly observed on the walls and are, contrary to the diatoms, involved in the formation of calcium carbonate speleothems. Whether fungi precipitate calcite actively as a result of their metabolism or secondly, as a response to favourable geochemical conditions, are still to be resolved. Several case studies on this phenomenon demonstrate the indirect influence of fungi on carbonate precipitation, e.g. on corals in coral-reef formations [94] and on formations of multilayered bryozoan reefs of *Celleporaria* sp. called bryostromatolites [95]. Although fungi are chemoheterotrophs and thus are not able to form their own organic compounds and use atmospheric CO_2_ as nutrients, it has been confirmed that some cryptoendolithic fungi are able to fix CO_2_ from the atmosphere and incorporate it into cell material [96, 97]. In the biofilm community, fungi thrive on the organic mass produced by other microbial stone colonizers such as Actinobacteria, algae or cyanobacteria and on deposits of organic matter such as bird or bat excretes. Our discovery of two black yeasts in the speleothem forming biofilm supports earlier findings of Blazquez et al., who reported black fungi as secondary inhabitants of thick biogenic calcium oxalate crusts on granite [98]. Such crusts were created by bacteria and later inhabited, penetrated, and partly destroyed by the fungi.

The growth of fungi and their diversity on rock surfaces are influenced by several factors, mainly by temperature, availability of nutrients and water activity. Sterflinger compiled a list of the most-abundant fungal species inhabiting rocks [99]. In arid and semi-arid environments, the black yeasts and microcolonial fungi are dominant, usually epilithic but also endolithic, often closely associated with lichens. They include members of *Knufia* L.J. Hutchison & Unter., *Hortaea* Nishim. & Miyaji, *Lichenothelia* D. Hawksw., *Trimmatostroma* Corda, *Ulocladium* Preuss, but also some fast-growing hyphomycetes such as *Alternaria* Nees or *Cladosporium* Link. In the humid environments with low or moderate temperature the fungal community shifts to fast-growing hyphomycetes such as *Alternaria*, *Aphanocladium* W. Gams, *Aureobasidium* Viala & G. Boyer, *Cladosporium*, *Epicoccum* Link, *Phoma* Sacc., *Penicillium* Link, *Trichoderma* Pers. or *Verticillium* Nees that form extensive mycelia in the porous space of the stone. The fast-growing hyphomycetes often produce a range of organic acids in order to penetrate and dissolve the rock and their mycelia are not usually melanised.

Isolation of samples of the abundant biofilm covering ca. 25 m^2^ of the exposed granite wall in the Kungsträdgården metro station yielded several fungi belonging to various lineages of Ascomycota and Basidiomycota including the new monotypic genus *Bacillicladium* and the new species *Bradymyces graniticola* described in our study. Moreover, *B*. *graniticola* was the only fungus targeted by the primers used in the environmental biofilm samples. Although our primary focus was the slow-growing melanised members of the Chaetothyriales, based on the BLAST search of ITS rDNA sequences of obtained isolates, we identified several members of *Cladosporium* spp., which are one of the most common indoor and outdoor fungi, *Acremonium nepalense* originally isolated from rhizosphere of *Pinus* in Nepal [100], and *Penicillium expansum*, a very common species and the most prevalent post-harvest rot of apples and other fruits. An interesting find represents the basidiomycete yeast-like *Trichosporon* cf. *akiyoshidainum* originally isolated from bat guano in bat-inhabited caves in Japan [101]. Members of *Trichosporon* Behrend, sometimes called oleaginous yeasts for their ability to accumulate lipids, are widely distributed in nature while some are part of the human microbiota and can occasionally cause infections [102, 103]. Due to their capability to produce microbial lipids by biodegrading certain environmental contaminants, e.g. polycyclic aromatic hydrocarbons, methanol, butanol, and acetone, they can play an important role in biotechnologies in decontaminating polluted environments [104–106]. The inoculation of the biofilm samples on various media yielded, in fact, many more fast-growing fungi, whose identification and isolation into axenic cultures was beyond the scope of our study.

The ability to use polycyclic aromatic components as a carbon source including petroleum products such as oil and gasoline (common pollutants of urban environments), is well documented in some members of the Chaetothyriales [107]. In the cells of *B*. *graniticola* we observed numerous vacuoles containing hydrophobic substances, almost certainly lipids. This material is frequently released from cells by a slight pressure on the microscopic preparation; the released lipid droplets always accumulate forming larger droplets. However, the potential of *B*. *graniticola* to efficiently decompose lipids needs to be tested.

*Bacillicladium lobatum* and *Bradymyces graniticola*, both members of the Chaetothyriales, have in common with other rock-inhabiting fungi slow-growing melanised colonies, vegetative mycelium consisting mostly of moniliform hyphae growing through enteroblastic proliferation and the tendency of shifting to meristematic development in older parts of colonies. These characteristics probably developed in non-related fungi during the process of convergent evolution and adaptation to similar extreme conditions.

### A New Lineage in the Chaetothyriales

Maximum likelihood and Bayesian inference of the two multilocus datasets of the Chaetothyriales and Chaetothyriomycetidae in our study (Figs 3 and 4) yielded clade that includes *Bacillicladium* as sister to three strains isolated from cardboard-like construction material produced by arboricolous ants [16, 17]. This lineage is unrelated to the five families currently distinguished in the Chaetothyriales. Two unnamed lineages including rock-inhabiting fungi identified by Gueidan et al. (as lineages 2 and 3) in the Chaetothyriales and Chaetothyriomycetidae [8] proved unrelated to *Bacillicladium* clade. Such topology opens up the possibility that *Ba*. *lobatum* and the three ant-associated fungi represent a distinct family. However, the lack of data and low statistical support in the ML analyses of the three- and four-gene datasets do not yet substantiate the formal introduction of a new group at family rank. It may, however, be possible in the future with improved statistical support for such grouping and more representatives included, whose morphology and ecological preferences will give a complete picture.

Based on the BLAST search, ITS sequences of *Ba*. *lobatum* showed 96−99% similarity with 11 sequences of environmental samples whose DNA was extracted from subaerial biofilms on granitic historic buildings of a World Heritage Site (Santiago de Compostela, NW Spain) [108]. In the three-gene analysis of members of the Chaetothyriales, the 11 environmental samples and two strains of *Ba*. *lobatum* were identified as conspecific (Fig 3).

A close association of plants with ascomycetes and non-attine ants in a mutualistic symbiosis has been known for some time and described from tropical regions of Africa, America and Asia [15, 16, 109, 110]. With the aid of molecular sequence data, a whole new group of fungi cultivated by ants in domatia (nesting places of ants provided by host plants—myrmecophytes) and on carton walls of nests and runaway galleries has been ascertained [15–17]. These fungi are undescribed, positioned mainly in the Chaetothyriales (Eurotiomycetes), but also in the Capnodiales (Dothideomycetes). Voglmayr et al. confirmed that fungi from domatia and cartons play different roles in the fungus-plant-ant association, they differ in morphology and ecology and the domatia fungi seemed to be specific with respect to ant species [16]. Based on ITS, nuc18S and nuc28S sequence data, Nepel et al. distinguished four main lineages of ant-associated fungi in the Chaetothyriales [17], i.e., a domatia-symbiont clade, two clades with carton fungi, and a mixed clade containing both domatia symbionts and carton fungi. Only a few isolates were placed outside these four clades.

The three strains of ant-associated fungi related to *Bacillicladium* belong to such ‘outsiders’. They occupy a basal position in a clade containing the Trichomeriaceae with the nested carton clade and the mixed clade [17]. These three strains were isolated from carton built by the Neotropical arboreal ant *Azteca brevis* Forel endemic to Costa Rica and Nicaragua (strains T430 Tm2, T357 TmE) [17] and Paleotropical *Crematogaster* sp. from Thailand (M-Cre1-1) [16]. It has been observed that carton-isolates do not exhibit yeast-like growth in culture and do not form conidia on the carton, but reproduce primarily by hyphal growth and hyphal fragments *in vivo* [16]. Their dark-walled monilioid hyphae reinforce the walls of nests and runaway galleries.

*Bacillicladium* is unique in the Chaetothyriales by having variable morphology showing hyphal, meristematic and yeast-like growth *in vitro*. Unlike the ant-associated fungi from carton, *Ba*. *lobatum* forms yeast-like cells at early stages of colony development on all media. The filamentous and meristematic phases can later replace the yeast-like growth and displace the budding cells on the margin of the colony on MEA and PDA while on PCA the yeast-like growth remains a prevalent form (Fig 8). The hyphal growth is prominent especially on MEA and PDA. The colonies are conspicuously velvety with two types of aerial hyphae. The first type of hyphae are up to 200 μm long, composed of aseptate cells that may appear uniseptate, shorter and wider than in the second type, which is composed of significantly longer hyphae, up to 700 μm long, the cells are thinner and longer appearing multiseptate. A single nucleus is always located in these cells. The number and position of nuclei in hyphal compartments that appear uni- and multiseptate were assessed under fluorescence microscope by staining heat-killed hyphae with propidium iodide. The septa are obscure, apparently discontinuous and function rather as cell wall reinforcements than true septa, i.e., the longer the cell are, the more of such structural reinforcements they contain. Apart from hyphae, small, usually 1–4-celled, ellipsoidal to globose, melanised fragments are formed singly or in chain *in vitro* probably taking part in propagation.

The majority of well-established genera in the Chaetothyriales can be distinguished by possessing distinct, branched conidiophores or, at least, distinct conidiogenous cells incorporated into vegetative hyphae. Members of the Chaetothyriales growing on rock surfaces mostly lack a yeast-like budding stage. Rock-inhabiting members of *Knufia* and *Bradymyces* of the Trichomeriaceae typically produce endoconidia, which are lacking in *Bacillicladium*. In contrast, *Neophaeococcomyces* of the same family represents another extreme where vegetative mycelium is lacking and the colony consists of a globular mass of chlamydospore-like cells.

The dominance of chaetothyrialean fungi in carton and domatia of arboricolous ants and the discovery of the new related fungus in the biofilm on the granite wall confirm their adaptability to extreme and even toxic environments. As mentioned earlier, members of the Chaetothyriales can utilise polycyclic aromatic hydrocarbons [107, 111–113], whose dominant sources in the environment are from human activity. Aromatic compounds, which are produced by metapleural and mandibular glands of ants, though toxic to various organisms, have antibacterial and antifungal properties [114–116] and obviously could be tolerated and even metabolized by symbiotic chaetothyrialean fungi [16]. Considering the amount of undescribed ant-associated fungi, their distribution pattern in the Chaetothyriales and their relationships with other morphologically similar melanised rock-inhabiting fungi, it can be postulated that many more species could be equipped with tolerance to toxic environments than hitherto known.

### Bradymyces

The genus *Bradymyces* was introduced for asexually reproducing fungi characterized by slow growth *in vitro*, melanised moniliform hyphae, blastic proliferation, the formation of endoconidia and multicellular and muriform bodies, which may develop dark fragmented incrustations on their surface [49]. *Bradymyces* is ecologically undefined. The type species *B*. *oncorhynchi* was isolated from a cold-blooded animal while both strains of *B*. *alpinus* occur on exposed rock surfaces and *B*. *graniticola* was isolated from a speleothem forming biofilm on a granite wall. Other undescribed samples analysed in this study originate from stone surfaces.

The three species accepted in *Bradymyces* are morphologically very similar and are mostly distinguished by growth at 10, 17 and 25°C (Figs 10–12). The lack of morphological characters has prompted a search for characters at the RNA structural level of ITS1 and ITS2 (Figs 6 and 7) that could objectively differentiate these species and other related, though unnamed rock-inhabiting isolates (Su et al., unpublished) and environmental samples [108] of *Bradymyces* spp.

*Bradymyces* is placed in a robust clade of the Trichomeriaceae (Figs 3 and 4) amidst the leaf epiphytes *Neostrelitziana* and *Strelitziana* [10, 117], undescribed rock-isolates, and species of *Knufia* inhabiting various niches including exposed rock and plant surfaces or occasionally recognized as opportunists that can cause infections in healthy humans [76, 118, 119]. Members of the family predominantly inhabit rocks and plants as saprobes or epiphytes [10, 36, 76, 117, 118, 120–126], and occasionally behave as weak plant pathogens [127], but are rarely isolated from clinical samples [49, 119, 128]. While the majority of fungi placed in the Trichomeriaceae reproduce exclusively asexually and display various modes of conidiogenesis, only members of foliicolous *Trichomerium* Speg. and the lichenicolous species *Knufia peltigerae* (Fuckel) Réblová & Unter., form dark, setose ascomata containing fissitunicate saccate asci with septate hyaline ascospores [11, 129].

The recently described family Strelitzianaceae [10] including *Strelitziana* and *Neophaeococcomyces* is here treated as a synonym of the Trichomeriaceae. The type species of both genera were shown in our multilocus phylogenies (Figs 3 and 4) to be distantly related to each other and nested among other members of the Trichomeriaceae.

### The CBC Species Concept in *Bradymyces*

In order to more precisely position *B*. *graniticola* among members of *Bradymyces*, 19 ITS sequences of *B*. *alpinus* and *B*. *oncorhynchi* [49], *Bradymyces* spp. [108] and other strains and environmental samples determined for this study and available in GenBank (Su et al., unpublished) were used to study intraspecific variability using the CBC criterion. The ITS2 retains a common core structure with three hallmarks in its RNA transcript, which are evolutionarily constrained and universal among eukaryotes [130, 131]. These hallmarks, which have proven useful for proposing molecular taxonomic concepts [132], include four helices, a pyrimidine-pyrimidine mismatch in helix II and the occurrence of the YGGUY motif in helix III. The utility of ITS2 sequences in barcoding of fungi and other organisms and phylogenetics is undoubted [133].

The CBC species concept [50, 51, 134, 135], is a hypothesis based on the occurrence of compensatory base change (CBC) in the conserved helices II and III ITS2 secondary structure and is correlated with the biological species concept [136]. CBC is defined as a double-sided substitution that would fulfil the hydrogen bonding requirements for pairing [90, 137]. The CBC concept can be utilised for example in an analysis of environmental samples, whose wild types are unknown, in order to identify the minimal number of species based on ITS2 secondary structure. Based on crossing experiments, Coleman introduced a species concept that involves CBC clades falling into one or very few sexually isolated so-called Z clades (= zygote or mating groups) [50]. These Z clades encompass groups of organisms producing compatible gametes that can form zygotes, but which are separated by various pre- and postzygotic mechanisms, thus may or may not produce fertile generations. Therefore, the Z clades contain one or few biological species. By the time the first CBC has appeared between two lineages, the clade may already contain one or more Z clades [50]. Coleman et al. showed on the example of the green alga *Chlamydomonas allensworthii* Starr et al. that within one morpho-species several biological species can be present [138]. On the other hand, the absence of a CBC between two organisms predicted that they belong to the same species with a probability of ~0.76 [52]. Species are further characterized by hCBCs and non-CBCs; however, their sole presence in the absence of CBC between two organisms indicates that they can theoretically interbreed. The rapidly evolving hCBCs and short-lived non-CBCs substitutions occur more frequently than CBCs [79] and may facilitate faster ecological adaptations of organisms followed by changes in morphology. These adaptations comprise specialization to new habitats, e.g. extreme environment, higher temperature, draught, nutrient-poor substrates, etc.

The expected number of CBCs between two sequences depends on their overall divergence [52]. A single CBC, which was encountered in helix II in the ITS2 among all analysed sequences of *Bradymyces* spp., suggests the existence of only two CBC clades, one of which is shown paraphyletic (Fig 5). These clades are further distinguished into six subclades characterized by the presence of unique hCBCs and non-CBCs, and they might correspond to reproductively isolated lineages, the so-called Z clades. All species of *Bradymyces* and the majority of genera in the Trichomeriaceae are exclusively asexual, or may also be cryptically sexual, but this is not explored. The low genetic divergence among all strains and environmental samples of *Bradymyces* analysed in our study demonstrates that they are closely related; in the absence of distinct phenotypes and mating studies, it may be difficult to recognize species boundaries based solely on the CBC concept.

The difference between *B*. *oncorhynchi* and *Bradymyces* sp. 2 of the same CBC clade, which is defined by the C = G pair, lies in a single hCBC in helix III in ITS1 (Fig 6, marked by asterisk) and in a sequence variation in the hairpin loops in helices III and V in ITS1 and helices II, III and IV in ITS2 (Fig 7). No difference occurs in the two most conserved helices II and III of ITS2 between the clinical isolate of *B*. *oncorhynchi* and three rock-inhabiting strains of *Bradymyces* sp. 2. For this pair of taxa, two conclusions may be possible: they represent two populations of the same species (Fig 5) suggesting that *B*. *oncorhynchi* is an opportunistic pathogen with rock surfaces as a possible natural reservoir, or they can be regarded as separate species, which needs to be proven by mating studies and comparative morphology.

The second CBC clade distinguished by the U-A pair from the previous CBC clade is shown to be paraphyletic in the phylogenetic analysis (Fig 5) and its four subclades III–VI can be clearly differentiated at the RNA structural level. All members of the subclades IV−VI are separated from *B*. *alpinus* (subclade III) by four unique hotspots, i.e., two non-CBCs in helices II and IV in the ITS1 and two hCBCs in helix III in the ITS2. The separated position of *B*. *alpinus* from other members of the same CBC clade in the phylogram and occurrence of unique hCBC events in ITS2 could be suggestive of ongoing speciation and specialization to new niches. The divergence between *B*. *graniticola*, *Bradymyces* sp. 1 and sp. 3 has no grounds in base changes in the 2D structure. They all belong to the same CBC clade and there are no unique hCBC or non-CBC substitutions in ITS1 and ITS2 that would further characterize them. The only difference lies in a primary sequence variation in the hairpin loop of helix V in ITS1 in *Bradymyces* sp. 1 and a single nucleotide substitution in a junction region in ITS2 in *B*. *graniticola*. However, the major difference between the speleothem biofilm isolates on one hand and rock-inhabiting strains of *Bradymyces* sp. 1 and environmental sample of *Bradymyces* sp. 3 on the other hand, lies in the absence of six base pairs and a hairpin loop in helix III in ITS1 (Fig 6). The event that led to the shortened helix III in its right arm and replacement of the original hairpin loop is unique to fungi isolated from the speleothem forming biofilm. Subclade four (Fig 5) is introduced as a new *Bradymyces* species in this study. A missing part of the helix III was observed also for *Bradymyces* sp. 4 (subclade VI) of the same CBC ‘U-A’ clade. One G = C pair is absent from the left arm of helix III in ITS1 and it also lacks one hCBC in helix III ITS1 (Fig 6). No further base changes could be identified in the ITS2 secondary structure of this rock-inhabiting environmental sample and its siblings from subclades IV and V. The ITS sequence of *Bradymyces* sp. 4 is shorter by ten nucleotides than in other members of *Bradymyces*. However, these gaps correlate with a conserved stem structure and rather suggest sequencing errors.

In our study we recognize three species of *Bradymyces*, which were studied in culture, *B*. *alpinus* and *B*. *oncorhynchi* [49] and *B*. *graniticola* (this study). The delimitation and a possible further treatment of monophyletic sibling subclades illustrated in Fig 5 containing other isolates of *Bradymyces* is suggested based on the CBC theorem and occurrence of hCBC and non-CBC types of substitutions.

## Supporting Information

S1 Table List of fungal names, isolate information and GenBank and EMBL accessions of *Bacillicladium*, *Bradymyces* and related taxa of the Chaetothyriomycetidae. Accession numbers in bold were generated for this study. Taxa indicated with an asterisk (*) represent environmental samples.
(DOCX)
