# Supplementary material for: From the Tunnels into the Treetops: New Lineages of Black Yeasts from Biofilm in the Stockholm Metro System and Their Relatives among Ant-Associated Fungi in the Chaetothyriales
Source: PLoS One. 2016 Oct 12;11(10):e0163396. doi: 10.1371/journal.pone.0163396 (PMC5061356; doi:10.1371/journal.pone.0163396)
Supplement: S1 Table — Accession numbers in bold were generated for this study. Taxa indicated with an asterisk (*) represent environmental samples. (DOCX) [file pone.0163396.s001.docx]

**S1 Table. List of fungal names, isolate information and GenBank and EMBL accessions of *Bacillicladium*, *Bradymyces* and related taxa of the Chaetothyriomycetidae**.
Accession numbers in bold were generated for this study. Taxa indicated with an asterisk (*) represent environmental samples.

| **Taxon** | **Source** | **Type** | **ITS** | **LSU** | **SSU** | ***rpb*2** | ***rpb*1** | **β-tubulin** | ***mcm*7** |
| --- | --- | --- | --- | --- | --- | --- | --- | --- | --- |
| *Aphanophora eugeniae* | CBS 124105 | T | FJ839617 | FJ839652 |  |  |  | KC455221 |  |
| *Arthrocladium caudatum* | CBS 457.67 | T | **LT558701** | **LT558701** | **LT558701** |  | **LT558711** | **LT558710** | **LT558717** |
| *Arthrocladium fulminans* | CBS 136243 | T | KT337439 | KT337444 |  |  |  |  |  |
| *Arthrocladium tardum* | CBS 134919 |  | KT337441 | KT337446 |  |  |  |  |  |
| *Arthrocladium tardum* | CBS 127021 | T | KT337440 | KT337445 |  |  |  |  |  |
| *Arthrocladium tropicale* | CBS 134926 | T | KT337442 | KT337447 |  |  |  |  |  |
| *Bacillicladium lobatum* | CCF 5200 | T | **LT558703** | **LT558703** | **LT558703** |  |  |  | **LT558718** |
| *Bacillicladium lobatum* | CCF 5199 |  | **LT558702** | **LT558702** | **LT558702** |  |  |  | **LT558719** |
| *Bacillicladium lobatum** | C3_38 |  | KU579748 |  |  |  |  |  |  |
| *Bacillicladium lobatum** | C3_69 |  | KU579779 |  |  |  |  |  |  |
| *Bacillicladium lobatum** | C3_449 |  | KU580159 |  |  |  |  |  |  |
| *Bacillicladium lobatum** | C3_1410 |  | KU581118 |  |  |  |  |  |  |
| *Bacillicladium lobatum** | C3_1648 |  | KU581356 |  |  |  |  |  |  |
| *Bacillicladium lobatum** | C3_1800 |  | KU581507 |  |  |  |  |  |  |
| *Bacillicladium lobatum** | C3_1897 |  | KU581604 |  |  |  |  |  |  |
| *Bacillicladium lobatum** | C3_2247 |  | KU581950 |  |  |  |  |  |  |
| *Bacillicladium lobatum** | C3_2357 |  | KU582059 |  |  |  |  |  |  |
| *Bacillicladium lobatum** | C3_2511 |  | KU582212 |  |  |  |  |  |  |
| *Bacillicladium lobatum** | C3_2698 |  | KU582398 |  |  |  |  |  |  |
| *Bradymyces alpinus* | CCFEE 5493 | T | HG793052 | GU250396 | GU250354 |  | **LT558712** | LN589970 | **LT558721** |
| *Bradymyces alpinus* | CCFEE 5478 |  | HG974249 |  |  |  |  |  |  |
| *Bradymyces oncorhynchi* | CCF 4369 | T | HG426062 | HG426063 | HG426064 |  | **LT558713** | HG426060 | **LT558720** |
| *Bradymyces graniticola* | CCF 5193 | T | **LT558704** | **LT558704** | **LT558704** |  | **LT558716** |  |  |
| *Bradymyces graniticola* | CCF 5194 |  | **LT558705** | **LT558705** | **LT558705** |  |  |  |  |
| *Bradymyces graniticola* | CCF 5195 |  | **LT558706** | **LT558706** | **LT558706** |  | **LT558715** |  |  |
| *Bradymyces graniticola* | CCF 5196 |  | **LT558707** | **LT558707** | **LT558707** |  |  |  |  |
| *Bradymyces graniticola* | CCF 5197 |  | **LT558708** | **LT558708** | **LT558708** |  | **LT558714** |  |  |
| *Bradymyces graniticola* | CCF 5227 |  | **LT558709** | **LT558709** | **LT558709** |  |  |  |  |
| *Bradymyces graniticola** | F6 |  | **KX179909** | **KX179911** | **KX179913** |  |  |  |  |
| *Bradymyces graniticola** | F6a |  | **KX179910** | **KX179912** | **KX179914** |  |  |  |  |
| *Bradymyces sp. 1* | CGMCC:3.17316 |  | KP174867 |  |  |  |  |  |  |
| *Bradymyces sp. 1* | CGMCC:3.17350 |  | KP174865 |  |  |  |  |  |  |
| *Bradymyces sp. 1* | CGMCC:3.17314 |  | KP174866 |  |  |  |  |  |  |
| *Bradymyces sp. 2* | CGMCC:3.17305 |  | KP174870 |  |  |  |  |  |  |
| *Bradymyces sp. 2* | CGMCC:3.17281 |  | KP174869 |  |  |  |  |  |  |
| *Bradymyces sp. 2* | CGMCC:3.17288 |  | KP174868 |  |  |  |  |  |  |
| *Bradymyces sp. 3** | C1_275 |  | KU579985 |  |  |  |  |  |  |
| *Bradymyces sp. 4** | C1_1050 |  | KU580759 |  |  |  |  |  |  |
| *Brycekendrickomyces acaciae* | CBS 124104 | T | FJ839606 | FJ839641 |  |  |  |  |  |
| *Camptophora hylomeconis* | CBS 113311 | T | KC455241 | EU035415 | KC455295 | KC455285 |  | KC455222 |  |
| *Capronia mansonii* | AFTOL-ID 656 |  |  | EF413604 | EF413603 | EF413606 | EF413605 |  |  |
| *Capronia semiimmersa* | MUCL 39979 |  | AF050260 | AF050260 | JN941210 | JQ027719 | JN989449 | EU514702 |  |
| *Celothelium cinchonarum* | F 17105 |  |  | DQ329020 |  |  |  |  |  |
| *Ceramothyrium carniolicum* | CBS 175.95 | T | KC455237 | KC455251 | KC455294 |  | FJ358364 |  |  |
| *Ceramothyrium podocarpi* | CPC 19826 | T | KC005773 | KC005795 |  |  |  |  |  |
| *Chaetothyrium agathis* | MFLUCC 12-C0113 | T | KP744437 | KP744480 |  |  |  |  |  |
| *Cladophialophora carrionii* | CBS 160.54 | T | AF050262 | FJ358234 | FJ358302 |  | FJ358366 |  |  |
| *Cladophialophora emmonsii* | IFM 52025 |  | AB109184 | AB100682 | KF155193 |  |  |  |  |
| *Cladophialophora hostae* | CPC 10737 | T |  | EU035407 |  |  |  |  |  |
| *Cladophialophora humicola* | CBS 117536 | T | EU035408 | KC809987 | KJ636038 |  | KJ636042 |  |  |
| *Cladophialophora minutissima* | CBS 121758 | T |  | KJ636047 | EF016370 |  | KJ636043 |  |  |
| *Cladophialophora modesta* | CBS 985.96 | T |  | FJ358236 | FJ358304 |  | FJ358368 |  |  |
| *Cladophialophora proteae* | CBS 111667 | T | EU035411 | EU035411 |  |  | KJ636044 |  |  |
| *Cladophialophora scillae* | CBS 116461 | T |  | EU035412 | KJ636040 |  | KJ636045 |  |  |
| *Cladophialophora sylvestris* | CBS 350.83 | T | EU035413 | EU035413 | KJ636041 |  | KJ636046 |  |  |
| *Cyphellophora laciniata* | CBS 190.61 | T | EU035416 | EU035416 | FJ358307 | KC455286 | FJ358370 | JQ766329 |  |
| *Cyphellophora fusarioides* | MUCL 44033 | T | KC455239 | KC455252 | KC455298 |  |  | KC455224 |  |
| *Cyphellophora olivacea* | CBS 123.74 | T | KC455248 | KC455261 | KC455304 |  |  | KC455231 |  |
| *Cyphellophora suttonii* | CBS 449.91 | T | KC455243 | KC455256 | KC455300 | KC455290 | JQ766404 | KC455226 |  |
| *Dermatocarpon miniatum* | AFTOL-ID 91 |  |  | AY584644 | AY584668 | DQ782863 | DQ782821 |  |  |
| *Dolabra nepheliae* | CBS 122120 |  |  | GU332517 |  |  | GU332521 |  |  |
| *Endocarpon pallidulum* | AFTOL-ID 661 |  |  | DQ823097 | DQ823104 | DQ840559 | DQ840552 |  |  |
| *Endocarpon pusillum* | AFTOL-ID 2279 |  |  | EF643754 | EF689837 |  | EF689756 |  |  |
| *Epibryon interlamellare* | M32 |  | EU940174 | EU940097 | EU940024 |  |  |  |  |
| *Epibryon plagiochilae* | M187 |  | EU940201 | EU940124 |  |  |  |  |  |
| *Epibryon diaphanum* | M122 |  | EU940178 | EU940101 | EU940028 |  |  |  |  |
| *Exophiala eucalyptorum* | CBS 121638 | T | KC455245 | KC455258 | KC455302 | KC455292 |  | KC455228 |  |
| *Geoglossum nigritum* | OSC 100009 |  |  | AY544650 | AY544694 | AY544740 | DQ471115 |  |  |
| *Granulopyrenis seawardii* | CBS 109025 |  |  | EF411062 | EF411059 | EF411065 |  |  |  |
| *Hyalocladosporiella tectonae* | CPC 23133 | T | KJ869142 | KJ86919 |  |  |  |  |  |
| *Knufia cryptophialidica* | DAOM 216555 | T | JN040501 | JN040501 | EF137364 |  |  |  |  |
| *Knufia epidermidis* | CBS 120353 | T | EU730589 | FJ355954 | FJ355953 |  |  |  |  |
| *Knufia marmoricola* | CCFEE 5886 | T | KP791779 | KR781067 |  |  |  |  |  |
| *Knufia petricola* | CBS 726.95 | T | AJ244275 | KC978741 |  |  |  |  |  |
| *Knufia perforans* | CBS 885.95 | T | AJ244230 | FJ358237 | EF137365 |  |  |  |  |
| *Metulocladosporiella musae* | CBS 161.74 | T | AY186199 | DQ008161 |  |  |  |  |  |
| *Metulocladosporiella musicola* | CBS 110960 | T | DQ008127 | DQ008159 |  |  |  |  |  |
| *Neophaeococcomyces aloes* | CPC 21873 | T | KF777182 | KF777234 |  |  |  |  |  |
| *Neophaeococcomyces catenatus* | CBS 650.76 | T | AF050277 | AF050277 | FJ358316 |  | FJ358379 |  |  |
| *Neostrelitziana acaciigena* | CPC 24873 | T | KR476730 | KR476765 |  |  |  |  |  |
| *Phaeomoniella effusa* | CBS 120883 | T |  | GQ154618 | GQ154639 |  |  |  |  |
| *Phaeomoniella chlamydospora* | CBS 23974 |  |  | AB278179 |  |  |  |  |  |
| *Phaeomoniella prunicola* | CBS 120876 | T |  | GQ154616 | GQ154637 |  |  |  |  |
| *Phaeomoniella tardicola* | CBS 121757 | T |  | GQ154619 | GQ154640 |  |  |  |  |
| *Phaeosaccardinula ficus* | MFLUCC 10-0080 | T | HQ895840 | HQ895837 |  |  |  |  |  |
| *Phialophora verrucosa* | MUCL 9760 |  | AF050281 | EF413615 | EF413614 |  | EF413616 | EU514714 |  |
| *Polyblastia viridescens* | CG 608 |  |  | EF643771 | EF689855 |  | EF689774 |  |  |
| *Pyrenula pseudobufonia* | AFTOL-ID 387 |  |  | AY640962 | AY641001 | AY641068 | DQ840558 |  |  |
| *Pyrgillus javanicus* | AFTOL-ID 342 |  |  | DQ823103 | NG_013194 | DQ842009 | DQ842010 |  |  |
| *Rhinocladiella anceps* | CBS 181.65 |  | EU041805 | EU041862 | AY554292 | DQ840564 | DQ840557 |  |  |
| *Rhynchostoma minutum* | UPSC 2337 |  |  |  | AF242268 |  |  |  |  |
| *Strelitziana africana* | CBS 120037 | T | DQ885895 | DQ885895 |  |  |  |  |  |
| *Strelitziana australiensis* | CBS 124778 | T | GQ303295 | GQ303326 |  |  |  |  |  |
| *Strelitziana malaysiana* | CPC 24874 | T | KR476731 | KR476766 |  |  |  |  |  |
| *Thelidium pyrenophorum* | AFTOL-ID 2250 |  |  | EF643782 |  |  | EF689785 |  |  |
| *Trichoglossum hirsutum* | KH 03232003-1 |  |  | AY544653 | AY544697 | DQ470881 | DQ471119 |  |  |
| *Trichomerium deniqulatum* | MFLUCC 10-0884 | T | JX313654 | JX313660 |  |  |  |  |  |
| *Trichomerium foliicola* | MFLUCC 10-0078 | T | JX313655 | JX313661 |  |  |  |  |  |
| *Trichomerium gloeosporum* | MFLUCC 10-0087 | T | JX313656 | JX313662 |  |  |  |  |  |
| *Veronaea botryosa* | CBS 254.57 | T | EU041816 | EU041873 | JN856021 |  |  | JN112505 |  |
| *Verrucaria macrostoma* | AFTOL-ID 2261 |  |  | EF643799 | EF689874 |  | EF689801 |  |  |
| *Vonarxia vagans* | CBS 123533 | T | FJ839636 | FJ839672 | KC455310 | KC455293 |  | KC455236 |  |
| Chaetothyriales sp. (ant-associated) | M-Cre1-1 |  | HQ634631 | HQ634631 |  |  |  |  |  |
| Chaetothyriales sp. (ant-associated) | T357 TmE |  | KF614872 | KF614872 |  |  |  |  |  |
| Chaetothyriales sp. (ant-associated) | T430 Tm2 |  | KF614813 | KF614813 |  |  |  |  |  |
| Chaetothyriales sp. (rock isolate) | BJ10108 |  | JQ061172 |  |  |  |  |  |  |
| Chaetothyriales sp. (rock isolate) | BJ10112 |  | JQ061171 |  |  |  |  |  |  |
| Chaetothyriales sp. (rock isolate) | BJ10118 |  | JN650519 |  |  |  |  |  |  |
| Chaetothyriales sp. (rock isolate) | TRN107 |  |  | FJ358253 | FJ358323 |  | FJ358386 |  |  |
| Chaetothyriales sp. (rock isolate) | TRN508 |  |  | FJ358265 | FJ358333 |  | FJ358398 |  |  |
| Chaetothyriales sp. (rock isolate) | TRN515 |  |  | FJ358266 | FJ358334 |  | FJ358399 |  |  |
| Chaetothyriales sp. (rock isolate) | TRN210 |  |  | FJ358255 | FJ358325 |  | FJ358388 |  |  |
| Chaetothyriomycetidae sp. (rock isolate) | A14 |  |  | FJ358268 | FJ358336 |  |  |  |  |
| Chaetothyriomycetidae sp. (rock isolate) | TRN242 |  |  | FJ358257 | FJ358326 |  | FJ358390 |  |  |
